# Stem cell exosome-loaded Gelfoam improves locomotor dysfunction and neuropathic pain in a rat model of spinal cord injury

**DOI:** 10.1186/s13287-024-03758-5

**Published:** 2024-05-20

**Authors:** Raju Poongodi, Tao-Hsiang Yang, Ya-Hsien Huang, Kuender D. Yang, Hong-Zhao Chen, Tsuei-Yu Chu, Tao-Yeuan Wang, Hsin-Chieh Lin, Jen-Kun Cheng

**Affiliations:** 1 Department of Medical Research, MacKay Memorial Hospital, Taipei, 10449 Taiwan; 2 Department of Anesthesiology, MacKay Memorial Hospital, Taipei, 10449 Taiwan; 3 Department of Medicine, MacKay Medical College, New Taipei City, 25245 Taiwan; 4 Institute of Long-Term Care, MacKay Medical College, New Taipei City, 25245 Taiwan; 5 Department of Pediatrics, MacKay Memorial Hospital, Taipei, 10449 Taiwan; 6 Institute of Clinical Medicine, National Yang Ming Chiao Tung University, Taipei, 11221 Taiwan; 7 Department of Pathology, MacKay Memorial Hospital, Taipei, 10449 Taiwan; 8 Department of Materials Science and Engineering, National Yang Ming Chiao Tung University, Hsinchu, 300093 Taiwan; 9 Center for Intelligent Drug Systems and Smart Bio-Devices (IDS 2 B), National Yang Ming Chiao Tung University, Hsinchu, 30068 Taiwan

**Keywords:** Exosomes, Spinal cord injury, Locomotory function, Nerve regeneration, Synapse formation, Glial scar, And neuropathic pain

## Abstract

**Background:**

Spinal cord injury (SCI) is a debilitating illness in humans that causes permanent loss of movement or sensation. To treat SCI, exosomes, with their unique benefits, can circumvent limitations through direct stem cell transplantation. Therefore, we utilized Gelfoam encapsulated with exosomes derived from human umbilical cord mesenchymal stem cells (HucMSC-EX) in a rat SCI model.

**Methods:**

SCI model was established through hemisection surgery in T9 spinal cord of female Sprague-Dawley rats. Exosome-loaded Gelfoam was implanted into the lesion site. An in vivo uptake assay using labeled exosomes was conducted on day 3 post-implantation. Locomotor functions and gait analyses were assessed using Basso-Beattie-Bresnahan (BBB) locomotor rating scale and DigiGait Imaging System from weeks 1 to 8. Nociceptive responses were evaluated through von Frey filament and noxious radiant heat tests. The therapeutic effects and potential mechanisms were analyzed using Western blotting and immunofluorescence staining at week 8 post-SCI.

**Results:**

For the in vivo exosome uptake assay, we observed the uptake of labeled exosomes by NeuN^+^, Iba1^+^, GFAP^+^, and OLIG2^+^ cells around the injured area. Exosome treatment consistently increased the BBB score from 1 to 8 weeks compared with the Gelfoam-saline and SCI control groups. Additionally, exosome treatment significantly improved gait abnormalities including right-to-left hind paw contact area ratio, stance/stride, stride length, stride frequency, and swing duration, validating motor function recovery. Immunostaining and Western blotting revealed high expression of NF200, MBP, GAP43, synaptophysin, and PSD95 in exosome treatment group, indicating the promotion of nerve regeneration, remyelination, and synapse formation. Interestingly, exosome treatment reduced SCI-induced upregulation of GFAP and CSPG. Furthermore, levels of Bax, p75NTR, Iba1, and iNOS were reduced around the injured area, suggesting anti-inflammatory and anti-apoptotic effects. Moreover, exosome treatment alleviated SCI-induced pain behaviors and reduced pain-associated proteins (BDNF, TRPV1, and Cav3.2). Exosomal miRNA analysis revealed several promising therapeutic miRNAs. The cell culture study also confirmed the neurotrophic effect of HucMSCs-EX.

**Conclusion:**

Implantation of HucMSCs-EX-encapsulated Gelfoam improves SCI-induced motor dysfunction and neuropathic pain, possibly through its capabilities in nerve regeneration, remyelination, anti-inflammation, and anti-apoptosis. Overall, exosomes could serve as a promising therapeutic alternative for SCI treatment.

**Supplementary Information:**

The online version contains supplementary material available at 10.1186/s13287-024-03758-5.

## Background

A growing number of individuals suffer from nerve-related injuries including traumatic brain injury, spinal cord injury (SCI), stroke, neurodegenerative diseases, and tumors. Among these disease entities, SCI is considered one of the most debilitating neurological diseases, which negatively impacts the patient’s quality of life [1, 2]. Severe SCI causes cell death, axonal loss, and demyelination and interrupts neuronal networks within the brain and periphery, resulting in loss of function [3]. Primary injury can be triggered through early mechanical stimuli, inflicting direct damage to spinal cord tissues. Furthermore, it can lead to neuronal death, nerve fiber breakage, edema, and hemorrhagic necrosis. These events are irreversible and occur after a nerve injury. Moreover, secondary nerve injuries include cavity formation, glial scarring, ischemia, hypoxia, inflammation, and excitotoxicity. These secondary nerve injuries can be managed medically, thus it has been the focus of SCI research [4].

Mesenchymal stem cell (MSC) implantation provides therapeutic effects in SCI [5, 6]; however, one of its limitations includes the difficulty in the direct transfer of MSCs into target tissues. The survival rate of implanted stem cells is very poor. Additionally, other possible complications may occur, such as tumor formation, immune rejection, and cell dedifferentiation [7–9]. Recently, research investigation on the effect of MSCs in tissue engineering has indicated that paracrine mechanisms may play a role in the action mechanism of MSCs in disease treatment, and exosomes had a significant role in this process [10, 11]. Exosomes are nanosized vesicles that are obtained during endosomal membrane invaginations and are key components of cellular paracrine secretion [12]. Moreover, exosomes are involved in the transportation of messenger RNAs (mRNAs), microRNAs (miRNAs), cytokines, and proteins; therefore, they play a key role in intercellular communication [13–15].

Exosomes embed more than 8000 proteins based on an online database. Apart from cell type-specific proteins, these exosomes also have common biomarkers, such as CD9, CD63, CD81, and TSG101 [16, 17]. Exosomes have specific surface ligands that can readily bind to target sites and regulate specific biological functions, such as intercellular signal transmission, angiogenesis, tumor-cell metastasis, and immune responses [18, 19]. Moreover, the therapeutic properties and adverse effects induced by exosomes remained better than those induced by stem cell transplantation [20]. Recently, numerous studies have focused on the application of stem cell-derived exosomes in tissue regeneration, immune response regulation, and regenerative medicine [21–23]. Importantly, after SCI, MSC exosomes can inhibit neuroinflammation and promote axonal regeneration [24, 25]. In particular, intravenous administration of human umbilical cord mesenchymal stem cell-derived exosome (HucMSCs-EX) can improve motor function and decrease cell death in a rat contusion SCI model [26]. Moreover, we use HucMSC-EX for this study due to their accessibility at clinical sites and potential therapeutic effects of stem cell exosomes.

For exosome delivery, certain biomaterials have been used as bioscaffold carriers in SCI treatment [27]. In this study, we selected the commercially available Gelfoam as an exosome carrier to bridge the spinal cord defect. This gelatin sponge has been widely used in medical industries due to its biodegradability and bio-compatibility in biological conditions [28], and it helps to control bleeding [29, 30]. Furthermore, the insertion of pure collagen filaments into the lesion site can induce axonal regeneration in the transected spinal cord and promote functional recovery [31]. Moreover, the gelatin sponge can be made into a spongy form to ensure cell attachment and it can cover an extensive surface area to treat the SCI [32]. In addition, Our previous investigations revealed that HucMSCs-EX, given intrathecally or locally, possess promising therapeutic effects in spinal nerve injury-induced pain [33, 34]. Although HucMSCs-EX has previously been studied for various SCI models, only very few reports found on the HucMSCs-EX-loaded Gelfoam for SCI-induced neuropathic pain (NP). Hence, there is an ample scope to explore more on the therapeutic effects of HucMSCs-EX-loaded Gelfoam in a rat SCI model. Herein, we report the HucMSCs-EX-loaded Gelfoam to treat the rat SCI model established through a unilateral hemisection surgery in the T9 spinal cord. The results showed that the implantation of HucMSCs-EX-encapsulated Gelfoam improves SCI-induced motor dysfunction and NP, possibly *via* its nerve regeneration, remyelination, glial inhibition, anti-inflammation, and anti-apoptosis capabilities.

## Materials and methods

### Animals and cell lines

All experiments were conducted using female Sprague-Dawley rats purchased from Bio LASCO Taiwan Co, weighing 225–250 g on the day of surgery. Before surgery, two rats were maintained in one plastic cage, and after surgery, the rats were maintained individually in plastic cages at room temperature with soft bedding and toys for environmental enrichment. The cages were maintained on a 12-h light and 12-h dark cycle with easy access to water and food. Sample sizes were calculated by estimating the smallest number of animals (n) needed to detect an arbitrarily chosen 60% increase in the mean of stabilized post-SCI behavioral data, with statistical significance set at *p* < 0.05 and power at 90% as our previous study [34]. The expected attrition or death of animals was adjusted for the final sample size using the following formula: (Corrected sample size = Sample size/ (1− [% attrition/100]) [35]. In total, 44 rats were divided into four groups, with 11 rats in each group. After surgery, animal behavior, infection, food intake, urine, and stool were monitored daily. Rats with severe body weight loss (> 20%) were euthanized and excluded from the experiment. The animals were euthanized in accordance with institutional guidelines and approved protocols. Euthanasia was performed using CO_2_ inhalation by trained personnel. The chosen method ensured rapid and humane euthanasia with minimal pain and distress to the animals. The experiment was performed according to a protocol (MMH-A-S-111-23, permitted on March 16, 2022) approved by the Institutional Animal Care and Use Committee and clinical trial (22MMHIS221e, Institutional Review Board, permitted on June 10, 2022) of MacKay Memorial Hospital, Taipei, Taiwan. Furthermore, PC12 cells were purchased from the Bioresource Collection and Research Centre (No. 60,019 and No. 60,048, Taiwan) for cell culture study (Additional file 1: Supplementary Materials and Methods 1 and 2). The work presented in this manuscript has been reported in line with the ARRIVE guidelines 2.0.

### Source and preparation of HucMSCs

HucMSCs were isolated from the umbilical cords obtained from 2 patients (source 1 for major study and source 2 for miRNA repeatability analysis) undergoing Cesarean section. These umbilical cords were washed with PBS under a sterile laminar flow cell culture hood and cut into 5cm^2^ pieces. Sections were cut lengthwise, blood vessels were removed, and sections were placed in 25-cm^2^ flasks. HucMSCs were cultured in adherent conditions and initially at 2500 cells/cm^2^ in 12-well plates with low-glucose Dulbecco’s Modified Eagle Medium (DMEM) and 10% fetal bovine serum (FBS) at 37 °C in humid air with 5% CO_2_. The culture medium was changed every 3 days. In this study, HucMSCs between passages 3 and 8 were prepared and used as previously described [31]. The cell viability was also greater than 95%.

### Isolation and purification of HucMSCs-EX

HucMSCs-EX were isolated and purified from human HucMSC culture supernatants as described in our previous study [36]. In brief, HucMSCs cultures were initially supplied at a density of 4 × 10^4^ cells/mL in 10 mL of low-glucose DMEM containing 10% FBS. When the cells reached 80% confluence (1.5 × 10^5^ cells/mL), the induction of exosome release was initiated by replacing the culture medium with serum-free low-glucose DMEM. Subsequently, these cells were cultured for an additional 48 h. Next, 300 mL of culture supernatant from 30 culture dishes (4.5 × 10^7^ cells/each) was collected and underwent a filtration process. Following filtration through a 0.45-µm filter to remove cell debris, a 0.22-µm filter was used to exclude apoptotic bodies. The culture supernatant was then concentrated using Vivaflow® 50R (SARTORIUS, Reference no. VF05H4, 100 KDa MWCO) [36] and Amicon® Ultra Centrifugal filter (Merk Millipore, Reference no. UFC9100, 100 KDa MWCO). The resulting exosome volume was between 1 and 2 mL, with exosome sizes ranging from 20 to 220 nm. The isolated exosomes were then stored at a temperature of -80 °C until they are ready to be used.

### HucMSCs-EX characterization assay

Multiple analyses were conducted to characterize the isolated HucMSC-EX, including the use of transmission electron microscopy (TEM), the NanoSight NS300 analyzer (Malvern Panalytical Ltd, Malvern, UK), BCA Protein Assay Kits (Thermo Fisher Scientific), and Western blotting for exosomal markers. For the TEM analysis, formvar-carbon-coated grids were placed on the surface of the exosome suspension (20 µL) with the coated side facing the suspension for 15 min. The grids were transferred to a wash buffer for 30 s (repeated 3 times). Subsequently, the grids were stained with a 2% uranyl acetate buffer for 15 min, followed by a 30-second wash repeated 3 times. The grids were left to air dry overnight at room temperature and were subsequently scanned using a TEM (JEM-1200EX II; JEOL Ltd, Tokyo, Japan) [37].

In another experiment, the NanoSight NS300 analyzer was used to analyze the size distribution of HucMSC-EX. These exosomes are generally observed to be between 30 and 150 nm in size and were quantified at 2–8 × 10^11^ particles/mL, originating from 4.5 × 10^7^ HucMSCs. Moreover, the protein concentration was determined to be 1.2 mg/mL using BCA Protein Assay Kits [36]. Additionally, Western blot was used to test the expression of CD9, CD63, CD81, Alix, β-actin, and calnexin in these isolated exosomes [36]. These antibodies were purchased from Cell Signaling Technology, Inc. and iReal Biotechnology, Inc. Next, HucMSC-EX obtained from 2 patients were concentrated by ExoQuick-TC (System Biosciences, SBI), and miRNA sequencing was performed using next-generation sequencing (NGS) (Additional file 1: Supplementary Materials and Methods 3).

### Establishment of rat SCI model

**Fig. 1:**
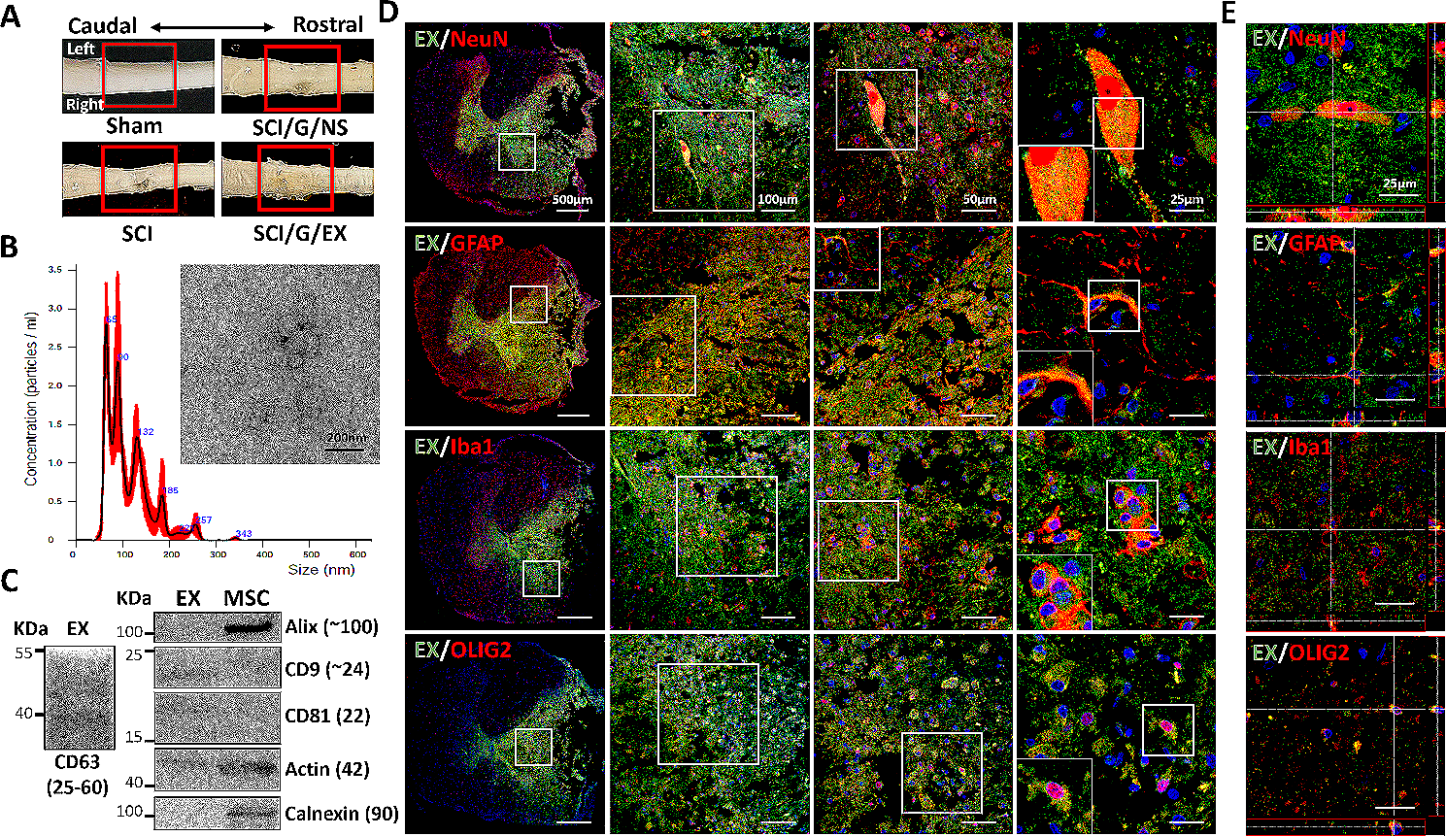
Treatment of spinal cord injury, characterization of HucMSC-EX, and in vivo exosome uptake by neurons and glial cells around the lesion site. **(A)** Overview of the lesion site in the spinal cord at week 8 post-surgery. The red box reflects the lesion area. **(B)**. Transmission electron microscope image of HucMSC-EX and analysis of exosome size distribution by NanoSight. **(C)** Western blot analysis of exosome markers. **(D, E)** Confocal microscopy images and orthogonal views (Z-stack projection) of internalized Exo-fluorescent green-labeled HucMSC-EX by neuronal nuclei (NeuN^+^), glial fibrillary acidic protein (GFAP^+^), ionized calcium-binding adaptor molecule 1 (Iba1^+^), and oligodendrocyte transcription factor 2 (OLIG2^+^) cells in the lesion site on day 3 post-surgery. The yellow spots indicate exosome uptake. The white box shows the magnification of the specific area. Scale bar: 500, 100, 50, and 25 μm as indicated. HucMSC-EX: human umbilical cord mesenchymal stem cell-derived exosome; SCI: spinal cord injury; G: Gelfoam; NS: normal saline; EX: exosome

Rats were anesthetized with isoflurane inhalation anesthesia and placed under a microsurgical apparatus in a prone position. Laminectomy was performed at the T7 vertebral level and a 2-mm right hemisection around the T9 spinal cord was created using a Micro Feather Ophthalmic Scalpel with a 15-degree angle blade (Electron Microscopy Sciences, catalog number 72045-15, Hatfield, PA) after hair shaving and sterilization with povidone-iodine. To standardize the lesions, the following steps were taken: (1) the blade was passed dorsally to ventrally three times to ensure all fibers were ablated, and (2) all surgeries were performed by one researcher to reduce variability [38]. Prior to surgery, the rats were randomized into four groups (sham, SCI, SCI/G/NS, and SCI/G/EX) using a True Random Number Generator from random.org. Furthermore, a commercially available absorbable Gelfoam (porcine gelatin sponge, Article no. MS0005, Johnson & Johnson, Canada), a hygienic, water-insoluble, flexible, and porous hemostatic with well-known characteristics material, was utilized in this study. This Gelfoam material was cut into 2 × 2 × 2 mm^3^ size and then immersed with exosome (10 µL, protein concentration: 1 µg/µL) or normal saline (control) in 1.5 mL Eppendorf tubes. Thereafter, the Gelfoam was placed and secured in the hemisected spinal cord area. Rats receiving laminectomy with or without SCI served as the SCI group or sham control. The graphic illustration of a right-sided T9 hemisection SCI and implantation of HucMSC-EX-loaded Gelfoam in the lesion site (Additional file 2: Fig. S1A). The representative image of a damaged spinal cord collected from different groups at week 8 after surgery is presented in Fig. 1A.

### Assessment of locomotor function and DigiGait analysis

For locomotor function evaluation, Basso–Beattie–Bresnahan (BBB) scoring was performed as previously described [39] 1–3 days before SCI and 1–8 weeks post-surgery for each animal. Gait data were collected using a DigiGait Imaging System (Mouse Specifics, Inc., USA), which is an advanced imaging system that digitally creates clear paw prints during a run on a treadmill [40]. The BBB score and gait behavior test were performed by examiners who were blinded to the group treatment. The animals were individually trained for 1 week prior to data collation. The training included a 5-min walk at different speeds within the range of the rats’ normal over-ground locomotion, ranging from 8 to 15 cm/s. Before testing, all rats were placed in a room near the testing area for 30 min for acclimation and then weighed. The animal on the stationary belt was initially moved at a rate of 8 cm/s and then gradually increased to 12 cm/s and maintained for 30 s. The DigiGait apparatus was used for each rat during the testing days. Each run was conducted for 3–5 min; thereafter, the machine was stopped, and the animal was transferred back to its cage.

### Von Frey filament and plantar thermal tests

The following tests were conducted at 8 weeks after surgery by an examiner blinded to the group treatment to display the nociceptive behaviors. The right hind paw withdrawal threshold (WT) in response to normally innocuous mechanical stimuli was evaluated using von Frey filaments as reported previously [33]. The foot withdrawal latency (WL) in response to noxious heat stimuli was measured using an Analgesia Meter apparatus (IITC/Life Science Instruments, CA, USA) as reported previously [33]. Four WLs obtained at 5-min intervals were averaged. Before each test, the rats were acclimatized for 30 min.

### Western blotting

To examine biomarkers of nerve regeneration, myelination, synapse formation, glial cell activation, neuroinflammation, and apoptosis around the SCI lesion site, Western blotting was performed at week 8 after surgery. Spinal cord tissues were homogenized in tissue lysis buffer containing protease inhibitors and phosphatase inhibitors (Sigma-Aldrich). Using NuPAGE Bis-Tris gradient gel (4–12%, Life Technologies, CA, USA), protein samples (20 µg/well) were separated and transferred into a polyvinylidene difluoride (PVDF) membrane. The PVDF membrane was incubated with primary antibodies against neurofilament 200 (NF200, 1:500, Sigma-Aldrich, MAB5266, mouse), myelin basic protein (MBP, 1:10000, Thermo Fisher Scientific, PA5-78397, rabbit), synaptophysin (1:1000, Millipore Sigma-Aldrich, AB9272, rabbit), postsynaptic density 95 (PSD95, 1:1000, Abcam, ab18258, rabbit), glial fibrillary acidic protein (GFAP, 1:1000, GeneTex, GTX89226, goat), growth-associated protein 43 (GAP43, 1:1000, Thermo Fisher Scientific, PA5-34943, rabbit), oligodendrocyte transcription factor 2 (OLIG2, 1:2000, Abcam, ab109186, rabbit), chondroitin sulfate 56 (CS56, 1:1000, Sigma-Aldrich, C8035-100UL, mouse), ionized calcium-binding adaptor molecule 1 (Iba1, 1:1000, Thermo Fisher Scientific, PA5-27436, rabbit), inducible nitric oxide synthase (iNOS, 1:1000, Thermo Fisher Scientific, 14-5920-82, rat), brain-derived neurotrophic factor (BDNF, 1:1000, Thermo Fisher Scientific, PA5-87530, rabbit), transient receptor potential cation channel subfamily V1 (TRPV1, 1:500, Abcam, ab203103, mouse), p75 neurotrophin receptor (p75NTR, 1:1000, Sigma-Aldrich, 07-476, rabbit), Cav3.2 (1:500, GeneTex, GTX54813, rabbit), phosphate-extracellular signal-regulated kinase 1/2 (pERK1/2, 1:1000, Cell Signaling 4377 S, rabbit), extracellular signal-regulated kinase 1/2 (ERK, 1:1000, Cell Signaling 4695 S, rabbit), Bcl-2-associated X protein (Bax, 1:1000, IR93-389, iReal, rabbit), and glyceraldehyde-3-phosphate dehydrogenase (GAPDH) (1:2000, Novus, NB300-221, mouse) after blocking with StartingBlock™ T20 (Thermo Fisher Scientific). Next, horseradish peroxidase-conjugated secondary antibody, developed in ECL solution, was used to incubate these blots, and the images were recorded using a cooled CCD system (LAS4000, Multi Gauge V3.0 software, Fujifilm, Japan). For the quantification of protein expression, the intensities of bands in each lane were measured using ImageJ software.

### Immunofluorescence study

To examine the expression of specific markers around the SCI lesion site at week 8 after surgery, a double immunofluorescence (IF) staining was conducted. The rats were perfused transcardially with normal saline buffer followed by 4% paraformaldehyde in 0.1-M phosphate buffer (0.08 M K_2_HPO_4_, 0.02 M NaH_2_PO_4_, pH 7.4) after sacrifice. Next, the spinal cord segments were separated and dehydrated in 15% and then 30% (w/v) sucrose in 0.1-M phosphate buffer. Spinal cord segment cryostats were cut into 20-µm sections. All tissue sections were placed directly on gelatin-coated slides. StartingBlock™ T20 was used to block non-specific binding. Two primary antibodies derived from different animal species were used for double staining, and the slides were simultaneously incubated. The primary antibodies against NF200 (1:300, Millipore, 2,986,162, mouse), MBP (1:400, Invitrogen, WD3255470D, rabbit), neuronal nuclei (NeuN) (1:400, Invitrogen, mouse), OLIG2 (1:200, Abcam, rabbit), GAP43 (1:500, Invitrogen, WB3204423C, rabbit), synaptophysin (1:300, Millipore, 3,015,857, rabbit), PSD95 (1:200, Invitrogen, WH331206, mouse), CS56 (1:400, Sigma-Aldrich, C8035, mouse) Iba1 (1:200, Invitrogen, rabbit), choline acetyltransferase (ChAT, 1:200, Millipore, 3,616,480, goat), and GFAP (1:400, GeneTex, 822,100,267, goat) were used. Alexa Fluor 488/594 conjugated goat or donkey anti-rabbit/mouse/goat (1:2000, Biotium, 14C0317, 15C0224, 16C0722, 17C0125, 21C0924) (1:5000, Biotium, 16C0722, 17C0130, 21C0615) were used as secondary antibodies. For the in vivo exosome uptake assay, the Exo-Glow™ protein labeling kit (System Biosciences, SBI, EXOGP300A) was used for HucMSCs-EX labeling before exosomes were soaked into Gelfoam. It aids in tracking and locating exosomes *via* covalently labeled internal EX proteins with green fluorescence. Sections on slides were mounted using Prolong™ Gold Antifade reagent with 4’,6-diamidino-2-phenylindole (2,305,157, Invitrogen) under coverslips. Using an Olympus FV300 confocal laser scanning microscope, the corresponding images were captured and processed with Adobe Photoshop 8.0 software (Adobe Systems, Mountain View, CA). In addition, the toluidine blue staining was performed to evaluate axons and myelin sheath formation (see Additional file 1: Supplementary Materials and Methods 4). All the images were used to measure the fluorescence intensity with ImageJ software.

### Data analysis and statistics

All data are presented as mean ± standard deviation (SD). Statistical significance was set at *p <* 0.05. Statistics were performed using GraphPad Prism version 7.0 (GraphPad Software, Inc., San Diego, CA). Two-way ANOVA was employed for the analysis of animal motor functions (*n* = 6–8 in each group), while one-way ANOVA was applied for the analysis of immunofluorescence intensity (*n* = 3 in each group) and Western blotting results (*n* = 5 in each group) followed by *post hoc* Tukey’s multiple comparisons tests.

## Results

### Characterization and in vivo uptake of HucMSC-EX

NanoSight analysis and TEM were employed to characterize the purified HucMSC-EX. The diameter distribution of HucMSC-EX ranged from 30 to 150 nm, and their morphology was observed to be a cup-shaped vesicle structure, as shown in Fig. 1B. Furthermore, several protein markers, CD9, CD63, and CD81, were detected in exosome samples by Western blotting, but calnexin was only observed in HucMSC samples (Fig. 1C). Moreover, the release profile of the exosomes from Gelfoam was analyzed using an in vitro release assay. Approximately 70% of HucMSC-EX was detected in the PBS buffer after 24 h and > 95% after 3 days (Additional file 2: Fig. S1B). These results indicated the characterization of our HucMSC-EX and their possible application in the treatment of rat SCI.

**Fig. 2:**
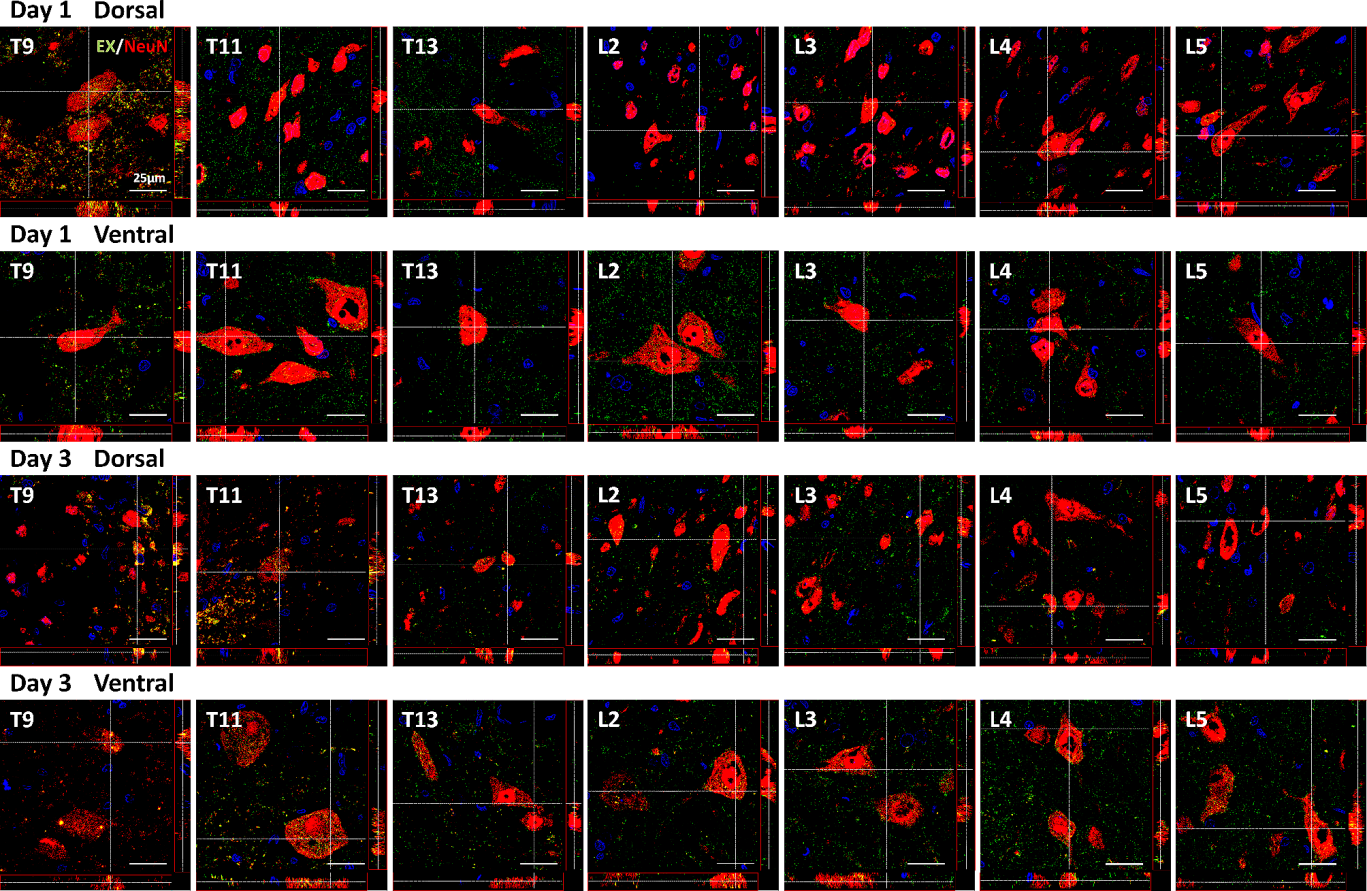
Temporal changes in the distribution of HucMSC-EX in the lesion site downstream area on days 1 and 3 post-SCI surgery. Orthogonal views (Z-stack projection) showed the uptake of Exo-fluorescent green-labeled exosome by neuronal nuclei (NeuN^+^) cells in the dorsal and ventral areas of the thoracic (T) and lumbar (L) regions. The yellow spots reflect the uptake of exosomes. Scale bar: 25 μm. HucMSC-EX: human umbilical cord mesenchymal stem cell-derived exosome; EX: exosome

Next, to analyze the fate of HucMSC-EX on day 3 after implantation, an in vivo exosome uptake assay was utilized. As demonstrated in Fig. 1D and Additional file 2: Fig. S1C, Exo-Green-labeled exosomes were observed in NeuN^+^, GFAP^+^, Iba1^+^, OLIG2^+^, and F4/80^+^ cells around the lesion site, indicating exosome uptake by neurons, glial cells, and macrophages. Notably, these exosomes can be detected in the cytoplasm but not in the nucleus, and the colocalization signals (yellow) were found inside these cells, as shown by Z-stack analysis (Fig. 1E). Additionally, we analyzed whether these internalized exosomes are distributed downstream of the lesion site. As shown in Fig. 2, on day 1 after implantation, Exo-Green-labeled exosomes were observed in NeuN^+^ cells within the dorsal and ventral areas of the spinal cord from T9 to T11. Compared with day 1, on day 3 after implantation, the exosomes progressively extended from T9 to L3 in the dorsal area and from T9 to L5 in the ventral area (Fig. 2). These results illustrated the distribution of HucMSC-EX after implantation and prompted us to examine the effects of exosomes on tissue repair, functional recovery, and neuronal protection after SCI.

### HucMSC-EX-loaded Gelfoam improves SCI-induced locomotor dysfunction and gait abnormality

**Fig. 3:**
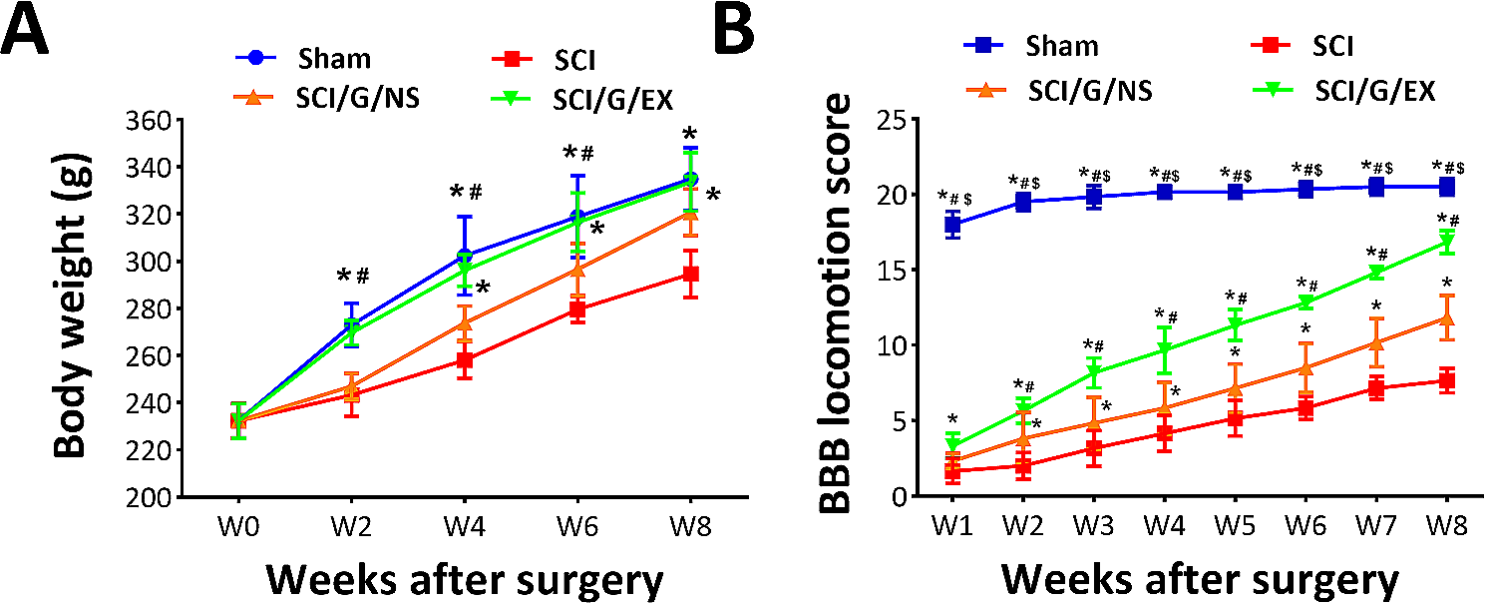
Locomotor function recovery in rats from week 1 to 8 after spinal cord injury. Temporal changes in animal’s body weight **(A)** and Basso-Beattie-Bresnahan (BBB) locomotion score **(B)**. Each data point represents the mean ± standard deviation. Group effect: significant, *p < 0.05 compared with the SCI group, ^#^*p* < 0.05 compared with the SCI/G/NS group, and ^$^*p* < 0.05 compared with the SCI/G/EX group using two-way analysis of variance (ANOVA) with *post hoc* Tukey’s multiple comparisons test (*n* = 6 in each group). SCI: spinal cord injury; G: Gelfoam; NS; normal saline; EX: exosome

We found that the body weight increase in the SCI/G/EX group was better than that in the SCI and SCI/G/NS groups over 8 weeks for the routine body weight measurement, implying a better food intake (Fig. 3A). We also tested whether HucMSC-EX treatment can improve SCI-induced locomotor dysfunction and gait deviation. The BBB score was used to evaluate the effects of HucMSC-EX on the improvement of open-field locomotor dysfunction. The BBB score in healthy rats was approximately 21, whereas laminectomy slightly affected the score at week 1 after surgery (sham group, 18 ± 0.89, mean ± SD) (Fig. 3B).

Next, we noted that the hemisection surgery can rapidly decrease the score to 1.66 ± 0.81 (SCI group, mean ± SD) at week 1 and maintain the lower status over 8 weeks, indicating a significant locomotor dysfunction. Nonetheless, the decline in the BBB score induced by SCI spontaneously recovered to 7.66 ± 0.81 over 8 weeks (SCI group). Progress was also observed in the SCI/G/NS (2.33 ± 0.51 for week 1 and 11.83 ± 1.47 for week 8) and SCI/G/EX (3.33 ± 0.81 for week 1 and 16.83 ± 0.75 for week 8) groups (Fig. 3B). The increasing trend of the BBB score from week 2 to week 8 in the SCI/G/NS group was better than that in the SCI group (*p <* 0.05), indicating a beneficial effect on motor recovery after Gelfoam implantation (Fig. 3B). Notably, the SCI/G/EX group had the highest BBB score from weeks 2 to 8, compared with the SCI and SCI/G/NS groups (*p <* 0.05). The BBB scores were 16.83 ± 0.75, 11.83 ± 1.47, and 7.66 ± 0.81 for the SCI/G/EX, SCI/G/NS, and SCI groups at week 8, respectively (Fig. 3B).

**Fig. 4:**
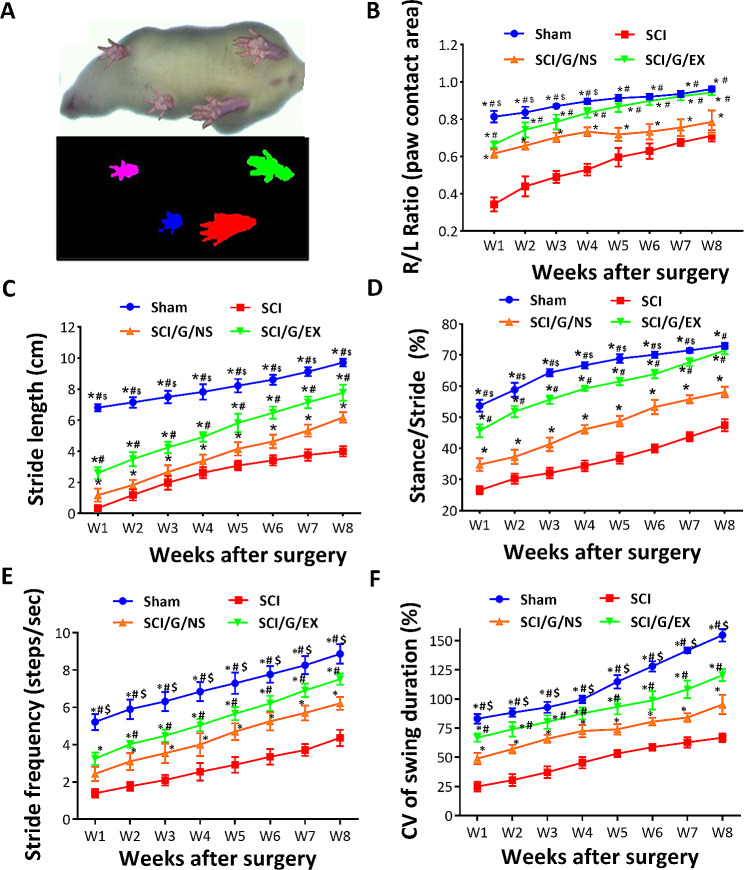
DigiGait walking analysis of rats from week 1 to 8 after spinal cord injury. **(A)** Representative image of the paw contact area. Temporal changes in right-to-left (R/L) hind paw ratio of the maximal paw contact area **(B)**, stride length **(C)**, stance/stride (time) ratio **(D)**, stride frequency (**E**), and coefficient of variation (CV) of the swing duration (**F**) of the right hind paw in four groups. Each data point represents the mean ± standard deviation. Group effect: significant, *p < 0.05 compared with the SCI group, ^#^*p* < 0.05 compared with the SCI/G/NS group, and ^$^*p* < 0.05 compared with the SCI/G/EX group using two-way ANOVA with *post hoc* Tukey’s multiple comparisons test (*n* = 6–8 in each group). SCI: spinal cord injury; G: Gelfoam; NS: normal saline; EX: exosome

Subsequently, to generate time-based gait parameters to study the effects of HucMSC-EX on the improvement of gait abnormalities in SCI rats, the DigiGait™ system was used. Although the DigiGait™ system computed more than 40 gait indices and postures [41], the trials also analyzed paw contact area, stride length, stride frequency, and swing duration variability (Fig. 4). Here, the stress-free experimental rats were allowed to walk to evaluate the accuracy and reproducibility of gait parameters. In healthy rats, the values of the maximum hind paw contact area (right-to-left ratio [R/L ratio]), stride length, and time ratio of stance/stride were 0.97 ± 0.01, 10.02 ± 0.26 cm, and 73.13 ± 1.38, respectively. At week 1, laminectomy (sham group) slightly affected these results (0.81 ± 0.03 for the R/L ratio, 6.81 ± 0.08 cm for stride length, and 53.7 ± 1.87 for stance/stride). Conversely, SCI can rapidly decrease gait stability (*p* < 0.05) and continue to decrease over 8 weeks, indicating gait abnormality (Fig. 4B and D). Similar to the BBB score, we noted that HucMSC-EX treatment significantly improved SCI-induced gait deviation (*p* < 0.05). Meanwhile, the beneficial effects produced by the implementation of exosome-loaded Gelfoam were better than those produced by the implantation of Gelfoam alone (*p* < 0.05, Fig. 4B and D). The R/L ratios (week 1/week 8) in each group were 0.81 ± 0.03/0.96 ± 0.01 (sham), 0.34 ± 0.04/0.71 ± 0.03 (SCI), 0.61 ± 0.02/0.78 ± 0.06 (SCI/G/NS), and 0.66 ± 0.01/0.94 ± 0.01 (SCI/G/EX) (Fig. 4B). In each group, the stride length (cm, week 1/week 8) was 6.81 ± 0.08/9.71 ± 0.08 (sham), 0.35 ± 0.06/4.00 ± 0.02 (SCI), 1.19 ± 0.15/6.19 ± 0.12 (SCI/G/NS), and 2.60 ± 0.13/7.77 ± 0.18 (SCI/G/EX) (Fig. 4C). The stance/stride (week 1/week 8) was 53.7 ± 1.87/73.00 ± 0.94 (sham), 26.57 ± 1.27/47.43 ± 1.97 (SCI), 34.77 ± 2.07/58.13 ± 1.69 (SCI/G/NS), and 45.67 ± 2.07/71.47 ± 1.22 (SCI/G/EX) in each group (Fig. 4D). The stride frequency (steps/s, week 1/week 8) in each group was 5.21 ± 0.42/8.87 ± 0.52 (sham), 1.40 ± 0.24/4.37 ± 0.42 (SCI), 2.43 ± 0.38/6.22 ± 0.34 (SCI/G/NS), and 3.25 ± 0.33/7.52 ± 0.30 (SCI/G/EX) (Fig. 4E).

Because an increased coefficient of variation (CV) of kinematic parameters has been reported to be associated with improved locomotor function in SCI rats [42], the CV of swing duration for each group was also analyzed. As shown in Fig. 4F, the SCI/G/EX group demonstrated an increased CV of swing duration compared with the SCI/G/NS and SCI groups (*p <* 0.05). The CV (%) of swing duration at week 1/week 8 in each group was 83.11 ± 4.05/154.68 ± 5.25 (sham), 25.00 ± 4.19/66.83 ± 5.25 (SCI), 49.00 ± 4.85/95.25 ± 8.26 (SCI/G/NS), and 66.83 ± 3.48/120.42 ± 5.22 (SCI/G/EX) (Fig. 4F), indicating that the increased variability of swing duration is correlated with better motor function recovery after SCI. Furthermore, our movie files show the improved locomotor function of exosome-treated rats at week 8 after surgery, compared with SCI rats (see Additional file 8: AVI files S1 to S4). In summary, these results indicated that treatment with exosomes could more effectively improve locomotor dysfunction and gait deviation than Gelfoam alone and SCI post-surgery.

### HucMSC-EX can facilitate neuronal protection and axonal regrowth around the lesion site after SCI

**Fig. 5:**
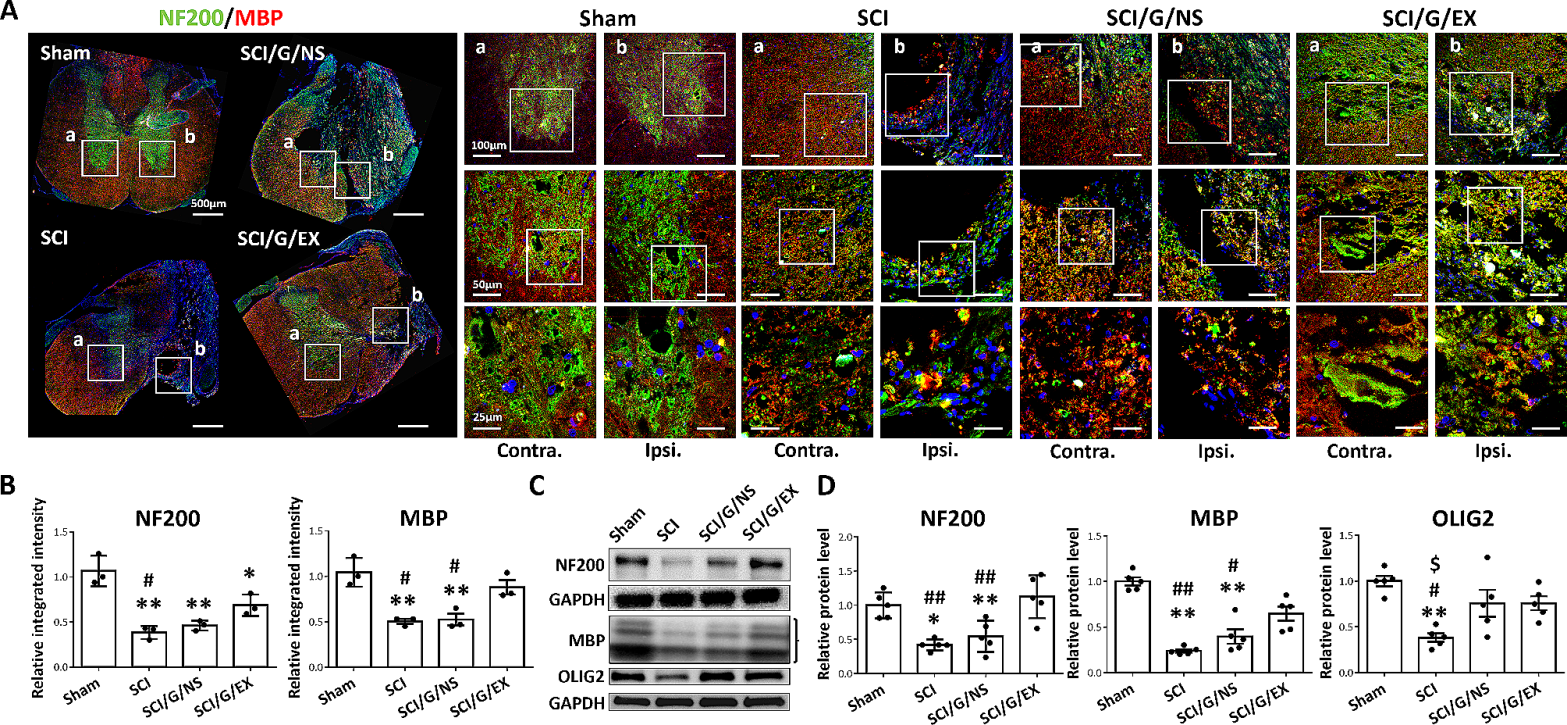
Implantation of HucMSC-EX-loaded Gelfoam promoted nerve regeneration and remyelination around the lesion site at week 8 after spinal cord injury. **(A, B)** Representative immunofluorescence images and relative integrated intensities of neuronal filament 200 (NF200, green) and myelin basic protein (MBP, red) around the T9 lesion site in four groups. The white box indicates the magnification of the specific area. Scale bar: 500, 100, 50, and 25 μm as indicated. *n* = 3 in each group. **(C, D)** Representative Western blots and relative protein levels of NF200, MBP, and oligodendrocyte transcription factor 2 (OLIG2) around the T9 lesion site. GAPDH was used as an internal control. Data are presented as mean ± standard deviation, taking the Sham group as 100%. *p < 0.05, **p < 0.01 compared with the Sham group, ^#^*p* < 0.05, ^##^*p* < 0.01 compared with the SCI/G/EX group, and ^$^*p* < 0.05 compared with the SCI/G/NS group through one-way ANOVA with *post hoc* Tukey’s multiple comparisons test, *n* = 5 in each group. HucMSC-EX: human umbilical cord mesenchymal stem cell-derived exosome; SCI: spinal cord injury; G: Gelfoam; NS: normal saline; Ipsi: ipsilateral; Contra: contralateral

We evaluated the status of neurons and axons, which play a vital role in SCI repair, to determine neuron protection and axon regrowth using IF staining and Western blot analysis. NF200 and MBP were used as markers for neuronal damage and axonal myelination after SCI [43]. In comparison with the sham group, the integrated intensities of NF200 and MBP decreased in the SCI group at week 8 after surgery, indicating neuronal loss and axon demyelination after SCI. However, the intensities of these two markers in the injured areas increased in the exosome group at 8 weeks post-SCI, as compared to the SCI group (Fig. 5A and B). Moreover, the results of Western blot analysis showed similar trends in the expression of NF200 and MBP in damaged spinal cord tissues (Fig. 5C and D), indicating that HucMSC-EX promoted neuronal and axonal protection following SCI. Notably, Gelfoam implantation alone did not markedly improve NF200 and MBP loss around the lesion site (Fig. 5A and B). Furthermore, in the results of IF staining, we observed that NF200 and MBP loss in the SCI and SCI/G/NS groups occurred bilaterally in the damaged spinal cord tissues (Fig. 5A, SCI and SCI/G/NS groups, a and b). In addition, OLIG2 is a marker for oligodendrocyte progenitor cells and is involved in the differentiation of oligodendrocytes [44]. Here, we found that SCI rats had decreased OLIG2 levels in the damaged spinal cord tissues compared with the sham group, indicating oligodendrocyte suppression by SCI (Fig. 5C and D). Gelfoam implantation reduced the suppressive effects of SCI, while treatment with exosomes did not further improve the decline in OLIG2 (Fig. 5C and D). This suggests that Gelfoam serves as a bio-scaffold for oligodendrocyte maintenance.

Next, toluidine blue staining was performed to assess the pathological changes in the myelin sheath within the damaged spinal cord tissues among different groups. The values (mean ± SD) of axon width (12.50 ± 4.20, 6.30 ± 2.50, 8.00 ± 3.70, and 11.00 ± 3.50 μm), fiber width (17.70 ± 5.00, 8.80 ± 3.20, 10.90 ± 4.40, and 16.00 ± 4.30 μm), and g-ratio (axon width/fiber width, 0.69 ± 0.07, 0.71 ± 0.08, 0.73 ± 0.09, and 0.68 ± 0.07) for the sham, SCI, SCI/G/NS, and SCI/G/EX groups are illustrated in Additional file 3: Fig. S1. According to these data, SCI can cause damage to nerve fibers and myelin sheath and cause smaller nerve fiber formation around the lesion site. Importantly, HucMSC-EX-loaded Gelfoam implantation improved these aberrant pathological changes more effectively than Gelfoam alone. These observations indicated a protective effect on nerve fibers and myelin sheaths after exosome treatment.

**Fig. 6:**
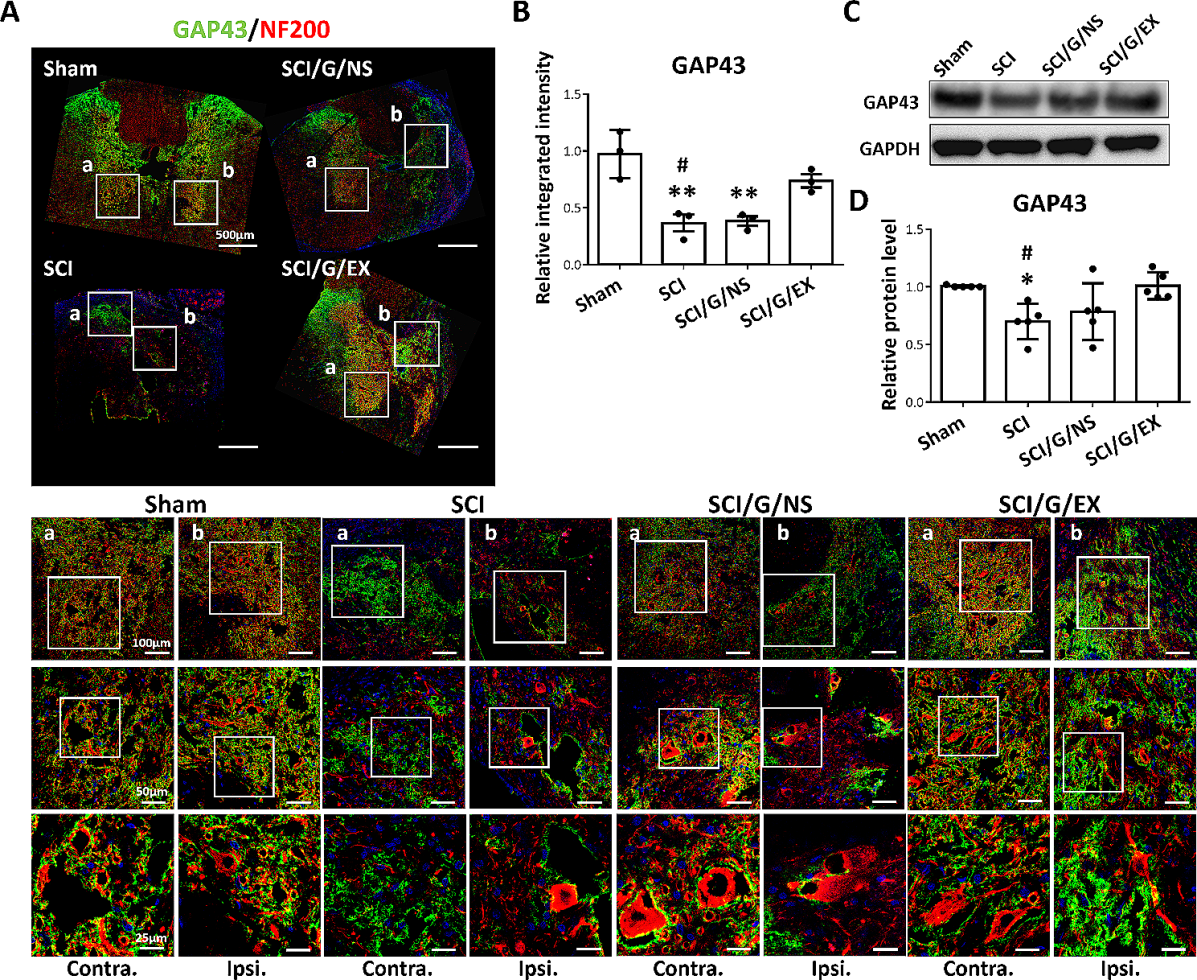
HucMSC-EX treatment decreased the spinal cord injury-induced growth-associated protein 43 (GAP43) loss around the lesion site at week 8 after surgery. **(A, B)** Representative immunofluorescence images of GAP43^+^ (green) and neuronal filament 200 (NF200, red) around the T9 lesion site in four groups. The white box reflects the magnification of a specific area. Scale bar: 500, 100, 50, and 25 μm as indicated. *n* = 3 in each group. **(C, D)** Representative Western blots and relative protein levels of GAP43 of the spinal cord around the T9 lesion site. GAPDH was used as an internal control. Data are presented as mean ± standard deviation, taking the Sham group as 100%. *p < 0.05, **p < 0.01 compared with the Sham group, and ^#^*p* < 0.05 compared with the SCI/G/EX group by one-way ANOVA with *post hoc* Tukey’s multiple comparisons test, *n* = 5 in each group. HucMSC-EX: human umbilical cord mesenchymal stem cell-derived exosome; SCI: spinal cord injury; G: Gelfoam; NS; normal saline; Ipsi: ipsilateral; Contra: contralateral

GAP43 is involved in axonal growth and generation of the neuronal network [43, 45]. Here, we noted that GAP43 is expressed close to NF200^+^ fibers in the spinal cord of the sham group (Fig. 6A, sham group, a and b). Meanwhile, the integrated intensities of GAP43 in the SCI group were lower than those in the sham group, indicating GAP43 loss by SCI (Fig. 6B). Conversely, the implantation of exosome-loaded Gelfoam reduced the SCI-induced loss of GAP43, whereas Gelfoam alone showed no significant effects (Fig. 6A and B). Moreover, the results of Western blot analysis showed similar trends in the expression of GAP43 in damaged spinal cord tissues (Fig. 6C and D).) Additionally, similar to NF200, GAP43 loss occurred bilaterally in the damaged spinal cord tissues in SCI rats (Fig. 6A, SCI group, a and b). Condensed fluorescence of GAP43 was also visualized around the lesion site of the exosome group, and GAP43 was partially co-expressed with NF200^+^ fibers (Fig. 6A, SCI/G/EX group, b), suggesting a beneficial effect on axonal protection and regeneration after exosome treatment.

In addition, we analyzed the pathological changes of α-motor neurons (ChAT^+^/NeuN^+^ cells) [46] in the ventral horn of the damaged spinal cord tissues through IF staining. At week 8 after surgery, it was observed that compared with the sham group, most of the ChAT^+^/NeuN^+^ cells were lost around the lesion site in the SCI and Gelfoam groups (Additional file 4: Fig. S1). Exosome treatment showed protective effects on motor neurons, as ChAT^+^/NeuN^+^ cells were visualized around the lesion site (Additional file 4: Fig. S1). According to the above-mentioned results, exosome-loaded Gelfoam exhibited more effective outcomes in reducing neuronal damage, preserving myelin sheath, and promoting axonal regrowth compared to Gelfoam alone.

### HucMSC-EX facilitates synaptic protection and synaptogenesis around the lesion site after SCI

**Fig. 7:**
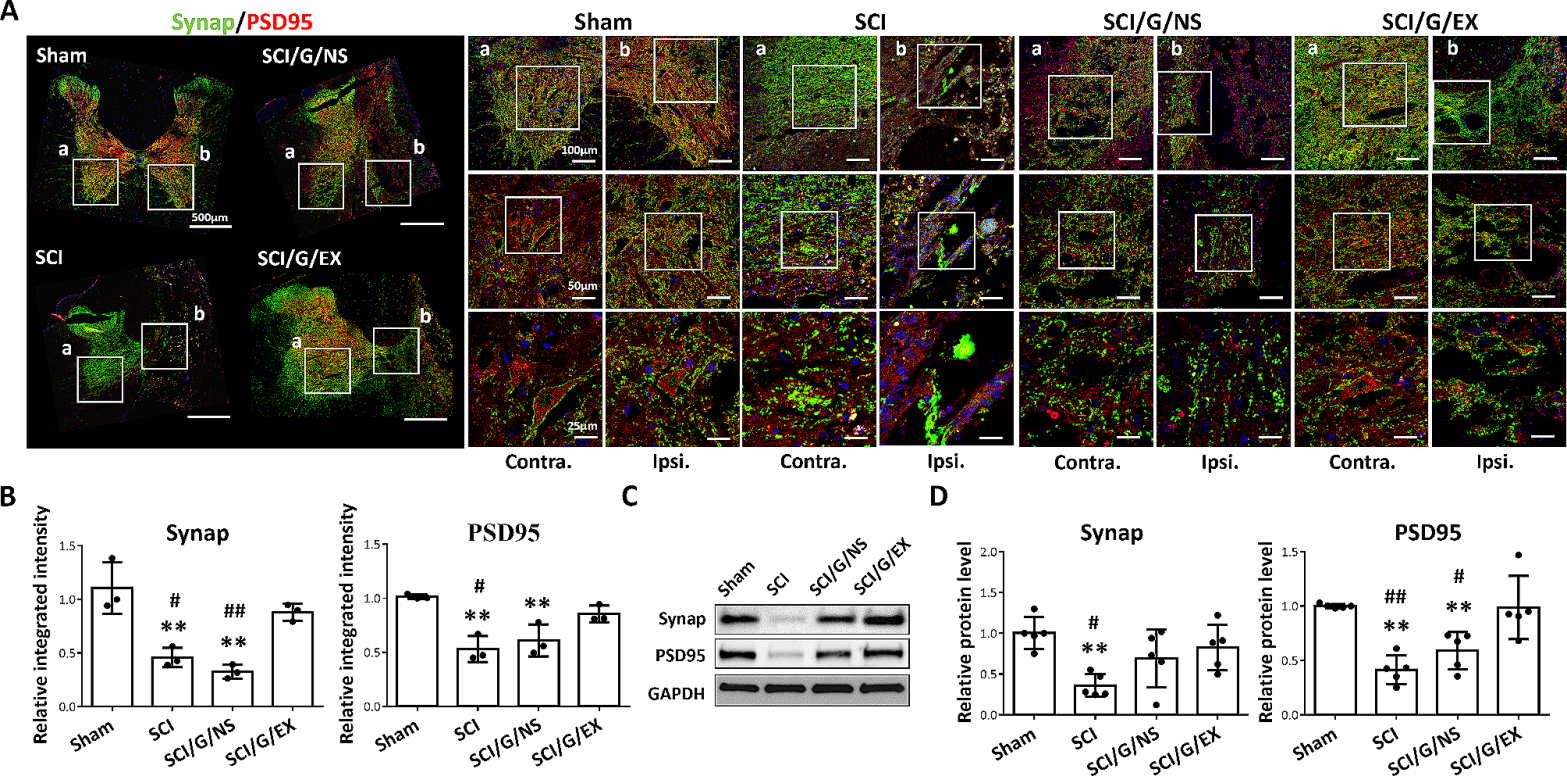
HucMSC-EX treatment attenuated the spinal cord injury-induced synapse loss at week 8 after surgery. **(A, B)** Representative Western blots and relative protein levels of synaptophysin (Synap, pre-synaptic marker, green) and postsynaptic density 95 (PSD95, post synaptic marker, red) around the T9 lesion site in four groups. The white box indicates the magnification of the specific area. Scale bar: 500, 100, 50, and 25 μm as indicated. *n* = 3 in each group. **(C, D)** Representative Western blots and relative protein levels of Synap and PSD95 around the T9 lesion site. GAPDH was used as the internal control. Data are presented as mean ± standard deviation, taking the Sham group as 100%. **p < 0.01 compared with the Sham group, ^#^*p* < 0.05, and ^##^*p* < 0.01 compared with the SCI/G/EX group through one-way ANOVA with *post hoc* Tukey’s multiple comparisons test, *n* = 5 in each group. HucMSC-EX: human umbilical cord mesenchymal stem cell-derived exosome. SCI: spinal cord injury; G: Gelfoam; NS; normal saline; Ipsi: ipsilateral; Contra: contralateral

The pre- and postsynaptic markers (synaptophysin and PSD95) were used to assess the pathological changes in synapses around the lesion site in rats with SCI to identify whether HucMSC-EX can promote synapse formation after SCI. In the sham group, synaptophysin and PSD95 were visible in the ventral horn of the spinal cord. In the meantime, PSD95 was encircled by synaptophysin (Fig. 7A, sham group, a and b). However, bilateral synaptic loss, reflected by decreased integrated intensities of synaptophysin and PSD95, as well as structural changes, was observed in the injured spinal cord tissues (Fig. 7A, SCI group, a and b, and Fig. 7B). Interestingly, exosome-loaded Gelfoam implantation improved these phenomena (Fig. 7A, SCI/G/EX group, b, and Fig. 7B). Notably, the effects of synapse formation after exosome treatment were better than those when Gelfoam was used alone (Fig. 7A and B). The results of Western blot analysis also showed that the levels of synaptophysin and PSD95 in the exosome group were higher than those in the SCI group (Fig. 7C and D). These results suggested that exosome treatment has positive effects on synaptic protection and formation.

### HucMSC-EX suppresses SCI-induced glial scar formation around the lesion site

**Fig. 8:**
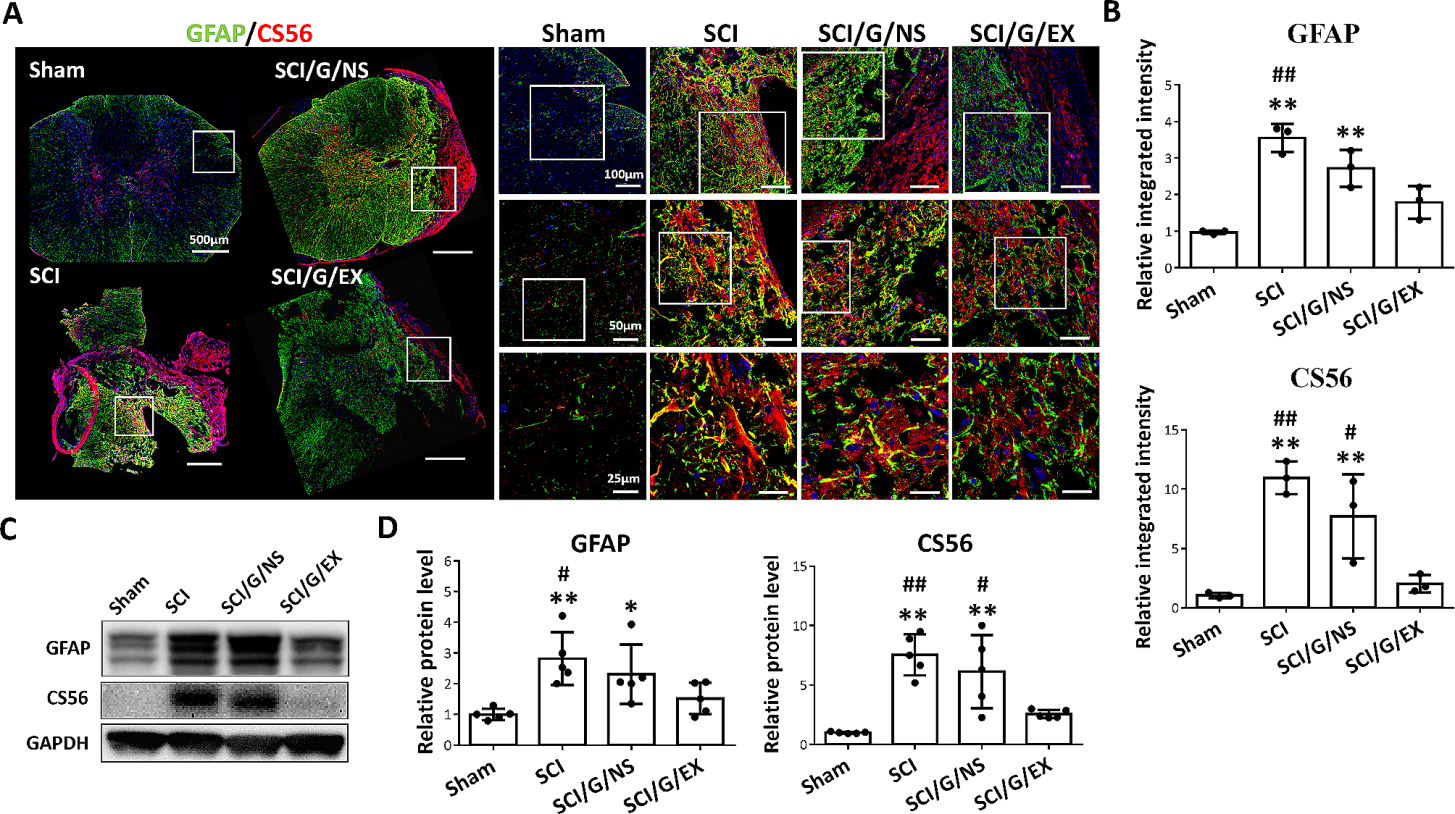
HucMSC-EX treatment decreased the spinal cord injury-induced astrocyte activation and CS56 production at week 8 after surgery. **(A, B)** Representative immunofluorescence images and relative integrated intensities of glial fibrillary acidic protein (GFAP, green) and chondroitin sulfate 56 (CS56, red) around the T9 lesion site in four groups. The white box indicates the magnification of a specific area. Scale bar: 500, 100, 50, and 25 μm as indicated. *n* = 3 in each group. **(C, D)** Representative Western blots and relative protein levels of GFAP and CS56 around the T9 lesion site. GAPDH was used as the internal control. Data are presented as mean ± standard deviation, taking the Sham group as 100%. *p < 0.05, **p < 0.01 compared with the Sham group, ^#^*p* < 0.05, and ^##^*p* < 0.01 compared with the SCI/G/EX group by one-way ANOVA with *post hoc* Tukey’s multiple comparisons test, *n* = 5 in each group. HucMSC-EX: human umbilical cord mesenchymal stem cell-derived exosome. SCI: spinal cord injury; G: Gelfoam; NS; normal saline

The HucMSC-EX effects on astrocyte activation and glial scar formation were evaluated 8 weeks after SCI surgery. As shown in Fig. 8A and B, the integrated intensities of GFAP and CSPG (CS56) were significantly increased in the SCI group, compared with the sham group, indicating astrocyte activation and glial scar formation post-SCI. However, implantation of exosome-loaded Gelfoam markedly reversed these results (Fig. 8A and B). In the results of Western blot analysis, exosome-loaded Gelfoam implantation revealed a significant effect in reducing GFAP and CSPG levels around the lesion site (Fig. 8C and D). In contrast, the implantation of Gelfoam alone showed a limited effect in this experiment. These results suggested that HucMSC-EX treatment can inhibit SCI-induced astrocyte activation, CSPG production, and glial scar formation around the lesion site.

### HucMSC-EX inhibits SCI-induced neuroinflammation and apoptosis

**Fig. 9:**
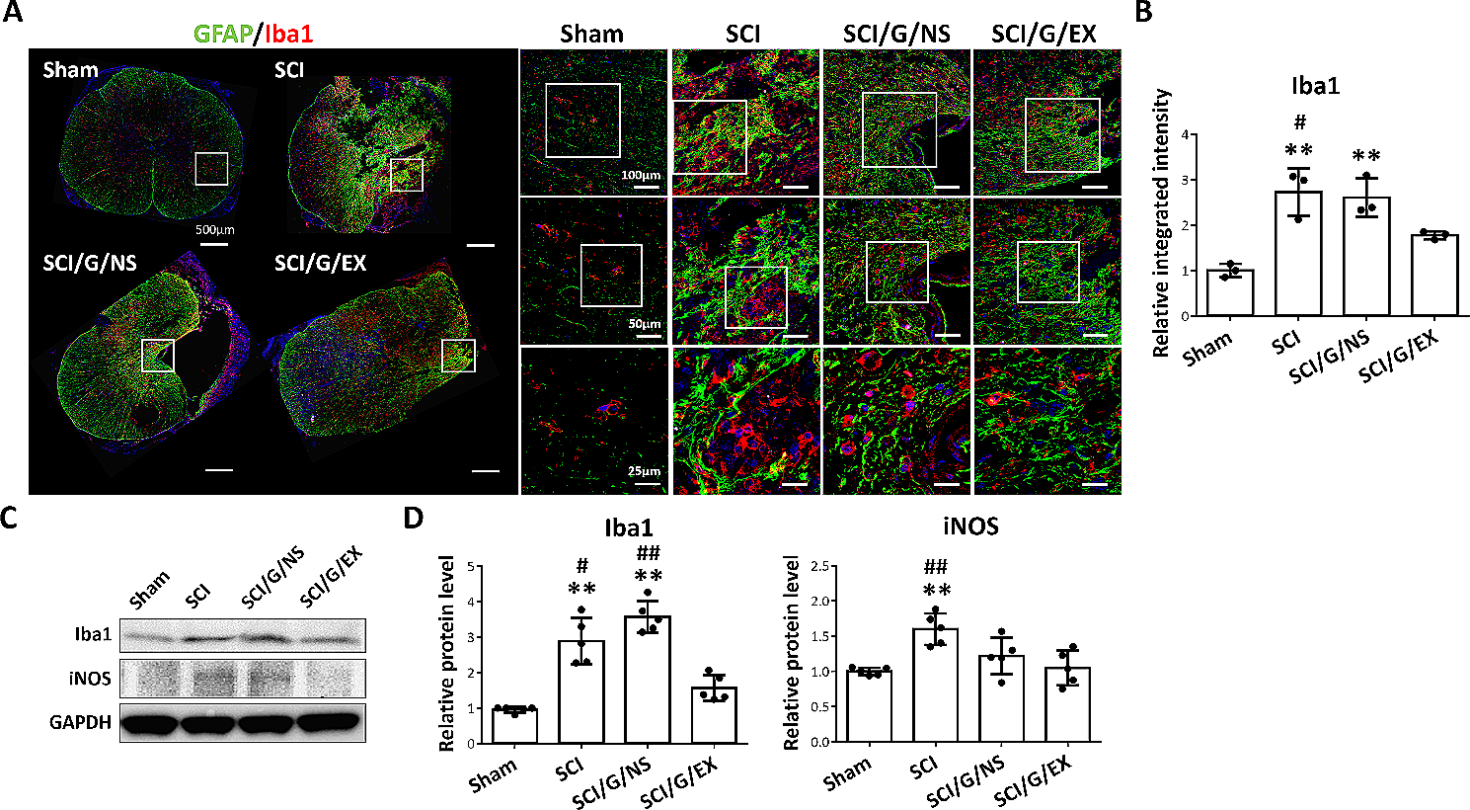
HucMSC-EX-loaded Gelfoam implantation reduced spinal cord injury-induced neuroinflammation at week 8 after surgery. **(A)** Representative immunofluorescence images of glial fibrillary acidic protein (GFAP, green) and ionized calcium-binding adaptor molecule 1 (Iba1, red) around the T9 lesion site in four groups. The white box indicates the magnification of the specific area. Scale bar: 500, 100, 50, and 25 μm as indicated. **(B)** Relative integrated intensity of Iba1^+^ staining in four groups. *n* = 3 in each group. **(C, D)** Representative Western blots and relative protein levels of Iba1 and inducible nitric oxide synthase (iNOS) around the T9 lesion site. GAPDH was used as an internal control. Data are presented as mean ± standard deviation, taking the Sham group as 100%. **p < 0.01 compared with the Sham group, ^#^*p* < 0.05, and ^##^*p* < 0.01 compared with the SCI/G/EX group through one-way ANOVA with *post hoc* Tukey’s multiple comparisons test, *n* = 5 in each group. HucMSC-EX: human umbilical cord mesenchymal stem cell-derived exosome; SCI: spinal cord injury; G: Gelfoam; NS: normal saline

SCI-induced neuroinflammation is associated with tissue damage and neurological dysfunction. Microglia are resident immune cells in the central nervous system (CNS). At week 8 after SCI, we tested the effect of HucMSC-EX on microglial activation. The SCI group demonstrated an increase in Iba1^+^ microglia and Iba1^+^ staining intensity around the lesion site compared with the sham group. However, the implantation of exosome-loaded Gelfoam (not Gelfoam alone) decreased GFAP^+^ and Iba1^+^ expression (Fig. 9A and B). Additionally, Western blot results demonstrated a significant reversal in the expression of GFAP and Iba1 proteins in spinal cord tissues induced by SCI in the HucMSC-EX treatment group (Fig. 9C and D). Meanwhile, exosome treatment suppressed the iNOS production induced by SCI around the lesion site (Fig. 9C and D). At week 8 after SCI, rats treated with exosomes demonstrated reduced levels of p75NTR and Bax around the lesion site compared with untreated rats (Additional file 5: Fig. S1). These results indicate that HucMSC-EX suppressed SCI-induced neuroinflammation and apoptosis.

### HucMSC-EX improves SCI-induced NP

**Fig. 10:**
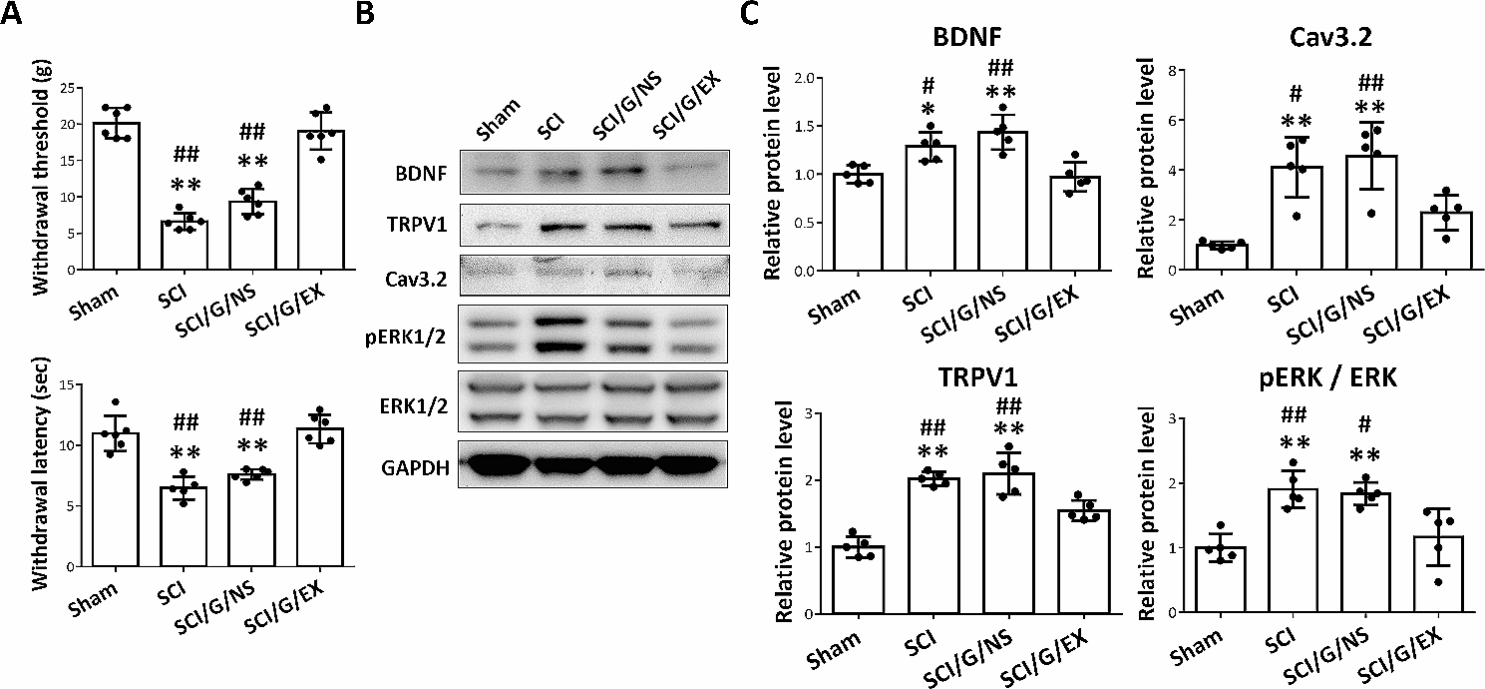
Implantation of HucMSC-EX-loaded Gelfoam alleviated spinal cord injury-induced neuropathic pain and pain-related protein upregulation at week 8 after surgery. **(A)** The withdrawal threshold and latency of the right hind paw in four groups. *n* = 6 in each group. **(B, C)** Representative Western blots and relative protein levels of brain-derived neurotrophic factor (BDNF), transient receptor potential vanilloid type-1 (TRPV1), Cav3.2 T-type calcium channel (Cav3.2), and phosphor-ERK1/2 (pERK1/2) around the T9 lesion site. GAPDH was used as an internal control. Data are presented as mean ± standard deviation, taking the Sham group as 100%. *n* = 5 in each group. *p < 0.05, **p < 0.01 compared with the Sham group, ^#^*p* < 0.05, and ^##^*p* < 0.01 compared with the SCI/G/EX group through one-way ANOVA with *post hoc* Tukey’s multiple comparisons test. HucMSC-EX: human umbilical cord mesenchymal stem cell-derived exosome; SCI: spinal cord injury; G: Gelfoam; NS: normal saline

We tested whether the HucMSC-EX could attenuate SCI-induced NP in this study. At week 8 after surgery, we observed that SCI rats had a reduced WT and WL in the right hind paw compared with the sham group (Fig. 10A). Meanwhile, the pain-related protein levels, such as BDNF, TRPV1, and Cav3.2 around the lesion site of SCI rats were also higher than that of the sham group (Fig. 10B). Conversely, exosome-loaded Gelfoam implantation (not Gelfoam alone) significantly reduced the upregulation of pain-related proteins and SCI-induced pain behaviors (Fig. 10). Moreover, Western blot analysis confirmed that SCI stimulates ERK phosphorylation, a marker for central sensitization [47] which contributes to NP. Subsequently, we observed the pERK/ERK expression level at week 8 induced by SCI was significantly reversed by the HucMSC-EX treatment group, not in Gelfoam alone (Fig. 10B and C). These results suggested that HucMSC-EX provides a promising therapeutic effect for treating SCI-induced NP.

### Identification of HucMSC-EX miRNAs

NGS was used to analyze the miRNA content in HucMSC-EX. We identified 563 types of miRNAs in exosomes derived from source 1, and the top 20 abundant miRNAs accounted for 59.5% of all the exosomal miRNA content (Additional file 6: Fig. S1**A**). To verify the repeatability of miRNA results, we examined the miRNA content from source 2, revealing that the top 20 abundant miRNAs accounted for 73.7% of all the exosomal miRNA content (Additional file 6: Fig. S1B). Notably, 55% of the top 20 abundant miRNAs (11 out of 20) overlapped between source 1 and 2 (Additional file 6: Fig. S1A and S1B).

Next, the top 20 abundant miRNAs from source 1 were further analyzed in five different biological domains (nerve regeneration, nerve remyelination, glial scar formation, cell death, and inflammation/pain), and the results are shown *via* a Venn diagram (Additional file 6: Fig. S1C). These five domains were closely correlated with tissue repair and neuronal protection after SCI. Furthermore, the miRNA sequences were analyzed using a combination of TargetScan and miRanda databases for the related target genes (Additional file 6: Table S1). The bioinformatics was also conducted using Gene Ontology (GO) and Kyoto Encyclopedia of Genes and Genomes (KEGG) pathway analyses, with the relevant biological processes and pathways demonstrated in Additional file 6: Fig. S1D and E. Notably, each miRNA participated in multiple biological processes and functions including neuronal development, cell migration regulation, cell proliferation, and apoptosis.

Moreover, we queried the top 20 abundant miRNAs from sources 1 and 2 in the treatment of SCI and NP using PubMed and presented the results in Additional file 6: Table S2. Among these miRNAs, we found that the overlapping miRNAs (miR-16-5p, miR-125b-5p, miR-21-5p, let-7a-5p, miR-29a-3p, let-7b-5p, let-7i-5p, miR-199a-3p, miR-199b-3p, miR-93-5p, and miR-26a-5p) are important for potential therapeutic effects, as relevant literature can be identified. In addition, Additional file 6: Table S3 displays the exosome-downregulated proteins, along with their possible related top 20 abundant miRNAs in HucMSC-EX from sources 1 and 2. These analytical results may establish relationships between exosomal miRNA and SCI treatment, offering insights for future mechanistic studies on HucMSC-EX.

### HucMSC-EX impacts on cell proliferation and neurite outgrowth in PC12 cells

We tested the effects of HucMSC-EX on cell growth and neurite formation in PC12 cells. First, Exo-Green-labeled exosomes were observed in the cytoplasm of PC12 cells, indicating the exosome uptake (Additional file 7: Fig. S1A). Next, the Cell Counting Kit-8 (CCK-8) assay showed that PC12 cells treated with exosomes (12.5, 25, and 50 µg/ml) exhibited an increased OD450 value compared to the control group (0 µg/ml), indicating a promotion in cell growth by the exosomes (Additional file 7: Fig. S1B). Furthermore, treatment of exosomes at 25 and 50 µg/ml markedly increased the neurite length in NGF-stimulated PC12 cells (Additional file 7: Fig. S1C). These results not only suggest the in vitro effects of HucMSC-EX on neuronal cell proliferation and neurite outgrowth but also provide positive evidence for our in vivo therapeutic effects.

## Discussion

SCI is a debilitating disease in which treatment is challenging and imposes a heavy disease burden on patients and their families. In this study, we provide evidence that HucMSC-EX can facilitate locomotor function recovery and improve NP in a T9 hemisected SCI model by inducing neuronal protection and axonal regeneration, suppressing glial scar formation and anti-inflammation, and reducing pain-related proteins. These findings suggest that exosomes derived from HucMSCs can serve as a potential therapeutic agent for improving SCI and its associated complications.

MSC transplantation has been shown to have a therapeutic effect in preclinical and clinical studies, but recent research suggests that exosomes are responsible for most of these effects. As a result, exosomes are gaining attention as a potential treatment for SCI and its complications [48]. Recipient cells take up exosomes, hence enhancing the transmission of information between microglia, astrocytes, neurons, and oligodendrocytes [49]. Indeed, after nerve injury, exogenous exosomes were found to play a role in the regulation of tissue repair. For example, microglia-derived exosomes have anti-oxidative properties, promote angiogenesis and aid in the recovery of neurological functions in SCI mice [49]. Interestingly, exosomes injected into the right gastrocnemius muscle of rats can be absorbed by the peripheral nerve endings and retrogradely transported into the dorsal root ganglia and ventral horn of the spinal cord [50]. Our previously studied intrathecally infused HucMSC-EXs also displayed a homing ability and improved nerve injury-induced NP [33]. In a contusive SCI model, MSC-derived exosomes can be internalized by the M2 microglia [51]. These findings indicated that exosome uptake by recipient cells is a crucial step in the induction of its physiological functions. In this study, we showed that (Exo-fluorescent green-labeled) exosomes can be observed around the T9 lesion site and incorporated into neurons, glial cells, and macrophages on day 3 after implantation (Fig. 1D and E, Additional file 2: Fig. 1C). These results suggested that the therapeutic effects of SCI observed in this study were associated with the internalized exosomes. Previous studies have confirmed that exosomes can persist at the site of injury for up to 14 days [52]. Our study highlights the extension of these exosomes from the T9 injury site to the lumbar area (Fig. 2). Therefore, depending on their target cells, HucMSC-EX could have diverse therapeutic effects on the damaged spinal cord.

Improvement of locomotor dysfunction and somatosensory abnormality are the aims of current SCI treatment. BBB score and gait analysis are vital indices to evaluate the effects of SCI treatment. Numerous studies reported on the BBB scores after exosome transplantation in animals [53]. For example, transplantation of neural precursor cells into SCI rats improves BBB score, stride length, and average speed at week 8 after surgery [54]. To reflect the gait abnormalities, the difference in paw parameters between the right and left hind paws was calculated. Previous studies have shown that the R/L ratio can be used to rule out the different influences of body weight and paw size [55]. According to our study, performing hemisection surgery on the right side of the T9 spinal cord can significantly impair the locomotion of the right hind paw. However, we found that treating SCI rats with exosomes resulted in improved locomotion and gait analysis. Specifically, at week 8 post-surgery, the exosome-treated rats showed increased scores on the BBB scale, longer stride lengths, and a higher R/L ratio of hind paw contact area (Figs. 3 and 4). These results indicated that HucMSC-EX provides promising therapeutic effects on locomotor dysfunction and gait abnormality after SCI.

Recently, several studies have shown that neuron protection and regeneration after SCI are associated with locomotor recovery and long-term prognosis. The decreased levels of NF200 and MBP were used to reflect nerve damage and axonal demyelination after nerve injury, while the increased levels of GAP43 can reflect the promotion of axon regrowth following treatment [43] [56]. . In this study, exosome treatment was observed to suppress the reduction of NF200 and MBP (Fig. 5), while increasing the levels of GAP43 (Fig. 6) around the lesion site. These results indicated the potential effects of exosomes in neuronal protection and axonal regeneration.

Additionally, toluidine blue staining was used for the assessment of pathological changes after nerve injury, including nerve morphology and axon myelination [57]. This staining technique provides data on axon width, fiber width, and the g ratio (axon width/fiber width) [58], enabling an understanding of the alterations in the myelin sheath structure following nerve injury [59]. Our toluidine blue staining results revealed that exosome treatment improved the pathological changes of the myelin sheath (increased axon width and fiber width, and decreased g-ratio) in the damaged spinal cord (Additional file 3: Fig. S1). Furthermore, oligodendrocytes can help reconstruct the injured myelin sheath [60]. Human spinal oligodendrogenic neural progenitor cells can promote functional recovery after SCI by facilitating axonal remyelination [61]. We found that implantation of Gelfoam alone and exosome-loaded Gelfoam had similar enhancing effects on OLIG2 in our SCI model (Fig. 5C and D). Overall, these findings indicate that HucMSC-EX treatment can induce neuron protection and axon regrowth after SCI.

Synapse formation is closely related to locomotor function recovery and long-term prognosis after SCI [62]. For instance, step training can upregulate positive synaptic genes and improve locomotor dysfunction in SCI rats [63, 64]. Induction of neurite outgrowth and synaptogenesis by RhoA inhibitors is associated with functional recovery in SCI rats [65]. Furthermore, bone marrow-derived MSCs displayed a therapeutic effect on the rearrangement of neural plasticity [66]. In this study, SCI caused synaptophysin and PSD95 loss around the lesion site, indicating synapse damage. Nevertheless, exosome treatment attenuated these phenomena (Fig. 7). These findings suggested that exosome treatment has beneficial effects on synapse formation and synapse protection after SCI. In addition, α-motor neurons (ChAT^+^/NeuN^+^ cells) are characterized by a large cell body and can innervate extrafusal muscle fibers and control muscle contraction [67]. Paralysis is a typical symptom of spinal motor neuron injury [67]. Implantation of stem cell-loaded hydrogel can facilitate α-motor neuron survival around the lesion site and functional recovery in SCI rats [68]. In this study, we showed that SCI rats had decreased ChAT and NeuN levels in the spinal ventral horn compared with the sham group (Additional file 4: Fig. S1), indicating spinal motor neuron destruction. However, restored motor function and ChAT^+^/NeuN^+^ cells were observed in exosome-treated SCI rats (Additional file 4: Fig. S1). Furthermore, the PC12 cell line is derived from a transplantable rat pheochromocytoma, and it is widely used as an in vitro model for neuronal differentiation, neuron regeneration, neuropharmacology, and toxicology [69]. Recently, several studies have cell proliferation, neurite outgrowth, and cell survival in PC12 cells [70]. In this study, we tested the effects of HucMSC-EX and found similar results on cell proliferation and neurite outgrowth in PC12 cells (Additional file 7: Fig. S1). Overall, these findings illustrate several positive effects induced by HucMSC-EX treatment, including neuronal protection, axon remyelination, synapse formation, and functional recovery after SCI.

Glial cells, such as astrocytes and microglia, mediate scar formation and neuroinflammation after SCI during tissue repair [71]. Astrocytes were rapidly activated and formed a dense border around the SCI lesion site, generally referred to as the glial scar. These scar-forming astrocytes may cooperate with certain inhibitory molecules and cause neuron regeneration failure [72]. CSPG, produced by scar-forming astrocytes, is one of the inhibitory molecules for nerve regeneration [73]. Bone marrow MSC-derived exosomes can reduce scar formation through A1 astrocyte and CSPG suppression [24]. In a murine model of ischemic stroke, miR-124 promoted nerve regeneration and functional recovery through astrocyte suppression [74]. In this study, we observed a decrease in GFAP and CSPG (CS56) around the lesion site, suggesting that exosomes inhibited astrogliosis, scar formation, and CSPG generation (Fig. 8). Similar to astrocytes, suppression of proinflammatory microglia can improve tissue damage and locomotor dysfunction. For instance, suppression of iNOS^+^ microglia around the lesion site can promote tissue repair and functional recovery in SCI mice [75]. The neuron-derived exosomes also decreased the Iba1 and iNOS levels in an SCI model [76]. Correspondingly, exosome treatment reduced the SCI-induced Iba1 and iNOS expression in the damaged spinal cord, indicating suppression of microglial activation in this study (Fig. 9). Since iNOS can be a marker for M1 microglia and A1 astrocyte, our finding of iNOS downregulation by HucMSC-EX suggests the glial polarization effect. Thus, HucMSC-EX treatment can reduce the activation of astrocytes and microglia around the lesion site, validating the potential effects of exosomes in suppressing SCI-induced neuroinflammation and glial scar formation.

Around 50% of patients with SCI suffer from NP [77]. Glial cells play a role in the development of nerve injury-induced NP. For instance, the expression of GFAP (an astrocyte marker) was increased in a chronic constriction injury model [78]. Additionally, inhibiting astrocyte activation and its associated signaling pathways has been shown to improve pain responses in a spinal nerve ligation model [79]. Furthermore, HucMSC-EX can induce analgesic effects in the L5/6 spinal nerve ligation pain model through its anti-inflammatory capacities, whether administered intrathecally or locally applied via a bio-scaffold [33, 34]. These findings prompted us to test the analgesic effects of exosomes in the SCI model. Similarly, a significant decrease in the WT and WL of the right hind paw at week 8 after SCI was observed, indicating the occurrence of mechanical allodynia and thermal hyperalgesia after SCI (Fig. 10A). Next, pain-related proteins, such as BDNF, TRPV1, and Cav3.2, have been demonstrated to mediate peripheral injury-induced pain and inflammation [80–82]. In this study, we showed that treatment of HucMSC-EX can suppress the expression of these pain-related proteins around the SCI lesion site (Fig. 10B and C). In addition, ERK expressed in the spinal dorsal horn neurons can be activated by noxious stimuli and serve as a marker for central sensitization [47]. In this study, the phosphorylated ERK (pERK) levels were increased in SCI rats compared with the sham group, indicating ERK activation by SCI (Fig. 10C). However, treatment of exosome-loaded Gelfoam can reverse these phenomena. Thus, we considered that HucMSC-EX may alleviate SCI-induced NP by modulating pain-related mediators.

SCI-induced cell death in neurons and glial cells worsens tissue damage and neurological deficits. Improvement in damaged spinal cords is crucial for treatment [83]. Apoptosis is a common form of cell death that can be induced *via* the activation of certain receptors, such as the tumor necrosis factor receptor, Fas ligand, and p75NTR [84]. In further studies, pro-apoptotic protein, Bax, caspase-3, and caspase-9 were upregulated, whereas the antiapoptotic protein Bcl-2 was downregulated after SCI [85]. Exosomes from various sources can hinder SCI-induced apoptosis and facilitate movement restoration by reducing proapoptotic proteins [85, 86]. . Although most of the apoptosis occurs in the early phase of SCI [83], some studies have found that the increase in proapoptotic proteins can persist for 4 weeks [85]. However, what remains consistent is that inhibiting apoptosis aids in the recovery of motor function after SCI [85]. In this study, we observed that exosome treatment reduced the levels of p75NTR and Bax around the T9 lesion site at week 8 after surgery (Additional file 5: Fig. S1), indicating potential antiapoptotic effects and contributions to functional recovery.

The Gelfoam can be used as a scaffold to support cell adhesion, tissue repair, angiogenesis, and anti-inflammation after SCI [87]. Gelfoam implantation into SCI rats can reduce the cavity area, glial scar formation, and CD68^+^ cells and increase the NF^+^ nerve fibers and Nissl^+^ cells [88]. In this study, we observed that Gelfoam alone can partially improve locomotor functions and gait impairments (Figs. 3 and 4) and axon degeneration (Additional file 3: Fig. S1) and suppress OLIG2 in SCI rats (Figs. 5 and 6). These results suggested that Gelfoam has a fundamental effect on SCI treatment due to its scaffold property.

The embedded miRNAs in exosomes are associated with the regulation of tissue repair following SCI. At least 150 types of miRNAs have been identified in exosomes derived from MSCs [89]. In this study, we observed that 55% of the top 20 miRNAs overlapped in two different sources, indicating repeatability and importance in these shared miRNAs (Additional file 6: Fig. S1 and Table S2 and S3). Furthermore, 11 overlapping miRNAs (miR-16-5p, miR-125b-5p, miR-21-5p, let-7a-5p, miR-29a-3p, let-7b-5p, let-7i-5p, miR-199a-3p, miR-199b-3p, miR-93-5p, and miR-26a-5p) were associated with the treatment of both SCI and NP and the literature can be found on PubMed (Additional file 6: Table S2). Interestingly, certain biomarkers for glial scar formation (GFAP and CSPG), neuroinflammation (Iba1 and iNOS), and pain-related molecules (BDNF, TRPV1, and Cav3.2) were predicted as targets of the top 20 abundant miRNAs (Additional file 6: Table S3). According to these bioinformatic results, the top 20 miRNAs found in HucMSC-EX may have a direct or indirect impact on the expression of biomarkers associated with SCI. These miRNAs seem to be closely linked to the therapeutic effects of exosomes for treating SCI. Overall, HucMSC-EX shows great potential for effectively treating SCI and offers a comprehensive and promising alternative.

### Limitations

Although this study elucidated the therapeutic effects of HucMSC-EX in the SCI model, some limitations remain. First, for the possible sexual differences [90], male rats should be examined in the future. Second, the potential therapeutic mechanisms, such as glial polarization, macrophage and neutrophil modulation [91] by HucMSC-EX in the early phase should also be studied. Within these limitations, the obtained results provide useful insights for clinical practice.

## Conclusion

HucMSC-EX treatment improves SCI-induced locomotor dysfunction and NP. This may be attributed to the effects of exosomes on neuroprotection, regeneration, remyelination, synaptogenesis, inhibition of glial cell activation, and pain-related protein expression. Thus, HucMSC-EX may be used as an alternative for SCI treatment. Furthermore, the detailed working mechanisms of exosomal miRNAs on the regulation of nerve regeneration and NP, including their roles in treating SCI, require future investigation. Efforts to enhance the yield and targeting ability of exosomes can aid the clinical application of HucMSC-EX in SCI management.

### Electronic supplementary material

Below is the link to the electronic supplementary material.

**Additional file 1**: Supplementary Materials and Methods.

**Additional file 2: fig. S1.**. **(A)** Graphical illustration of hemisection surgery at T9 spinal cord on the right side and implantation of exosome-loaded Gelfoam in the lesion site. **(B)**In vitro exosome release assay demonstrating the percentage of soaked exosomes released from the Gelfoam from day 1 to 7 in the 96-well plate. Data are presented as mean ± standard deviation (*n* = 3). **(C)** Confocal microscopy images and orthogonal views (Z-stack projection) of internalized Exo-fluorescent green-labeled HucMSC-EX by F4/80^+^ macrophages around the lesion site on day 3 post-surgery. The yellow spots indicate exosome uptake. Scale bar: 25 μm as indicated. HucMSC-EX: human umbilical cord mesenchymal stem cell-derived exosome; SCI: spinal cord injury; EX: exosome.

**Additional file 3: fig. S1**. HucMSC-EX treatment decreased spinal cord injury-induced axon degeneration around the lesion site at week 8 after surgery. **(A)** Representative histological images of toluidine blue staining around the T9 lesion site. Scale bar: 40 μm as indicated. **(B)** The axon width, fiber width, and g-ratio of four groups. The high-resolution image (100) was used to calculate the g-ratio (ratio of axon width to fiber width). Data are presented as mean ± standard deviation. *p < 0.05, **p < 0.01 compared with the sham group, ^##^*p* < 0.01 compared with the SCI/G/EX group, and ^$^*p* < 0.05 compared with the SCI/G/NS group through one-way ANOVA with *post hoc* Tukey’s multiple comparisons test. **(C)** The correlations between axon width, fiber width, and g-ratio are demonstrated. HucMSC-EX: human umbilical cord mesenchymal stem cell-derived exosome; SCI: spinal cord injury; G: Gelfoam; NS: normal saline.

**Additional file 4: fig. S1**. HucMSC-EX treatment improved the destruction of motor neurons around the lesion site at week 8 after spinal cord injury. **(A, B)** Representative immunofluorescence images and relative integrated intensities of choline acetyltransferase (ChAT, motor neuron marker, green) and neuronal nuclei (NeuN, red) around the T9 lesion site in four groups. The white box shows the magnification of the specific area. Scale bar: 100, 50, and 25 μm as indicated. *n* = 3 in each group. Data are presented as mean ± standard deviation, taking the Sham group as 100%. *p < 0.05 and **p < 0.01 compared with the sham group through one-way ANOVA with *post hoc* Tukey’s multiple comparisons test, *n* = 3 in each group. HucMSC-EX: human umbilical cord mesenchymal stem cell-derived exosome; SCI: spinal cord injury; G: Gelfoam; NS; normal saline; Ipsi: ipsilateral; Contra: contralateral.

**Additional file 5: fig. S1**. HucMSC-EX-loaded Gelfoam implantation attenuated spinal cord injury-induced apoptosis at week 8 after surgery. **(A, B)** Representative Western blots and relative protein levels of p75NTR and Bax around the T9 lesion site at week 8 after spinal cord injury. GAPDH was used as an internal control. Data are presented as mean ± standard deviation, taking the Sham group as 100%. *p < 0.05, **p < 0.01 compared with the sham group, and ^##^*p* < 0.01 compared with the SCI/G/EX group by one-way ANOVA with *post hoc* Tukey’s multiple comparisons test, *n* = 5 in each group. HucMSC-EX: human umbilical cord mesenchymal stem cell-derived exosome; SCI: spinal cord injury; G: Gelfoam; NS: normal saline.

**Additional file 6: fig. S1**. Identification of miRNA content of human umbilical cord mesenchymal stem cell-derived exosome (HucMSC-EX) through next-generation sequencing (NGS). **(A, B)** The top 20 abundant miRNAs of HucMSC-EX from two sources. **(C)** Distribution of the abundant top 20 miRNA in five relevant biological functional domains (nerve regeneration, nerve remyelination, glial scar formation, cell death, and inflammation/pain) through the Venn diagram. **(D, E)** The hot map of the biological process and Kyoto Encyclopedia of Genes and Genomes (KEGG) pathway analysis in the top 20 abundant miRNAs using ShinyGo (http://bioinformatics.sdstate.edu/go/).

**Additional file 6: table S1**. The top 20 abundant miRNAs of source 1 HucMSC-EX with their predicted target genes (retrieved from TargetScan and miRanda miRNA database) are categorized into five relevant biological functional domains: nerve regeneration, remyelination, glial scar formation, cell death, and inflammation/pain.

**Additional file 6: table S2**. References to support the potential therapeutic effects of the top 20 abundant miRNAs of source 1 and source 2 HucMSC-EX in spinal cord injury and neuropathic pain.

**Additional file 6: table S3**. The exosome-downregulated proteins in this study and their possible related top 20 abundant miRNAs in source 1 and source 2 HucMSC-EX.

**Additional file 7: fig. S1**. HucMSC-EX can promote cell proliferation and neurite outgrowth in PC12 cells. **(A)**In vitro exosome uptake assay. PC12 cells were divided into three groups: Group 1 is PC12 cells alone, Group 2 is PC12 cells with unlabeled exosomes, and Group 3 is PC12 cells with Exo-Green-labeled exosomes. **(B)** Effects of exosome on cell proliferation in PC12 cells. PC12 cells were treated with HucMSC-EX (12.5, 25, and 50 µg/ml) for 72 h. Cell Counting Kit-8 (CCK-8) was used to evaluate the effects of exosomes on PC12 cell proliferation. **(C)** Effects of exosome on neurite outgrowth in NGF-treated PC12 cells. PC12 cells were pretreated with HucMSC-EX (25 and 50 µg/ml) for 24 h and then stimulated by NGF (50 ng/ml) for another 5 days. *p < 0.05 and **p < 0.05. HucMSC-EX: human umbilical cord mesenchymal stem cell-derived exosome; NGF: neural growth factor.

**Additional file 8: AVI file S1**. Rat walking on a motorized transparent treadmill belt after spinal cord injury at week 8 after surgery.

**Additional file 8: AVI file S2**. DigiGait software program showed the rat walking and digital footprints in a rat spinal cord injury group at week 8 after surgery.

**Additional file 8: AVI file S3**. Rat walking on a motorized transparent treadmill belt after human umbilical cord mesenchymal stem cell-derived exosome treatment for spinal cord injury at week 8 after surgery.

**Additional file 8: AVI file S4**. DigiGait software program showed the rat walking and digital footprints after human umbilical cord mesenchymal stem cell-derived exosome treatment in rat spinal cord injury group at week 8 after surgery.

**Additional file 9:**

## Data Availability

The datasets during and/or analyzed during the current study are available from the corresponding author on reasonable request.

## Abbreviations

ANOVA: Analysis of variance
Bax: Bcl-2-associated X protein
BBB: Basso–Beattie–Bresnahan
BDNF: Brain-derived neurotrophic factor
ChAT: Choline acetyltransferase
CS56: Chondroitin sulfate 56
GAP43: Growth-associated protein 43
GFAP: Glial fibrillary acidic protein
GO: Gene Ontology
HucMSCs-EX: Human umbilical cord mesenchymal stem cell-derived exosome
Iba1: Ionized calcium-binding adaptor molecule 1
IF: Immunofluorescence
iNOS: Nitric oxide synthase
KEGG: Kyoto Encyclopedia of Genes and Genomes
MBP: Myelin basic protein
miRNAs: microRNAs
mRNAs: messenger RNAs
MSCs: Mesenchymal stem cells
NF200: Neurofilament 200
NGS: Next-generation sequencing
OLIG2: Oligodendrocyte transcription factor 2
PSD95: Postsynaptic density 95
PVDF: Polyvinylidene difluoride
SCI: Spinal cord injury
SD: Standard deviation
SNL: Spinal nerve ligation
TRPV1: Transient receptor potential cation channel subfamily V1
WL: Withdrawal latency
WT: Withdrawal threshold
