# Supplementary material for: Stem cell exosome-loaded Gelfoam improves locomotor dysfunction and neuropathic pain in a rat model of spinal cord injury
Source: Stem Cell Res Ther. 2024 May 20;15:143. doi: 10.1186/s13287-024-03758-5 (PMC11103960; doi:10.1186/s13287-024-03758-5)
Supplement: Supplementary file 1 — Additional file 1: Supplementary Materials and Methods. [file 13287_2024_3758_MOESM1_ESM.docx]

**Additional file 1: Supplementary Materials and Methods**

1. **Assessment of PC12 proliferation**

PC12 cells (6.5 × 10^4^ cells/ml) were seeded in collagen-type I-coated 24-well cell culture plates (Gibco, A10483-01) using low-serum medium (2% horse serum and 1% fetal bovine serum). After 12 hours, the medium was replaced with a complete culture medium (10% horse serum and 5% fetal bovine serum), and these PC12 cells were treated with human umbilical cord mesenchymal stem cell-derived exosome (HucMSC-EX, 12.5, 25, and 50 μg/ml) for 72 hours. Cell Counting Kit-8 (CCK-8) was used to evaluate the effects of exosomes on PC12 cell proliferation. Briefly, CCK-8 solution was added into each well and incubated at 37°C for 2 hours. The absorbance value at 450 nm (OD450) was determined by a microplate reader (SpectraMax® ABS Plus, Molecular Devices).

**Analysis of cell growth and neurite outgrowth**

PC12 cells (6.5 × 10^4^ cells/ml) were seeded in collagen-type I-coated 24-well cell culture plates using low-serum medium (2% horse serum and 1% fetal bovine serum) for 12 hours. Following pretreatment with HucMSC-EX (50 μg/ml), PC-12 cells were stimulated by nerve growth factor (NGF, 50 ng/ml) for 5 days. The medium was replaced every 2 days. For measurement of neurite length, 8-14 randomly selected fields in each well were captured by EVOS™ XL Core Imaging System (Thermo Fisher Scientific Inc.), and approximately 10 neurites in each field were used to calculate the average neurite length. Neurites exhibiting a length less than the diameter of the cell body were excluded from the calculation [1]. The average neurite length was measured and calculated using NeuronJ, a plugin in the ImageJ package, Fiji (version 1.54f, National Institutes of Health, Bethesda, MD, USA) [2].

1. **MicroRNA sequencing and library preparation for HucMSC-EX**

Total RNA (5 µL) was extracted from 200 µL exosomes and used as input material for the small RNA sample preparations. Then, the sequencing library was generated using QIAseq® miRNA Library Kit (QIAGEN, Germany). Previously, the first-strand cDNA was synthesized using oligo-dT and QIAseq miRNA NGS reverse transcriptase. After polymerase chain reaction amplification, the library was size selected using 170-200 bp by QIAseq beads. Then, the Qsep400 system was used to assess the quality of purified libraries. At that time, these qualified libraries were sequenced on the Illumina NovaSeq6000 system with sheared single-end 75 bp. These procedures were performed by Genomics, BioSci & Tech Co., New Taipei City, Taiwan. The software packages TargetScan (<http://www.targetscan.org/mmu_72/>) and Miranda (http://www.microrna.org/microrna/getDownloads.do) were used to identify miRNA binding sites and predict the gene targets of the abundant top 20 miRNAs.

**4. Toluidine blue staining**

Toluidine blue staining was performed to evaluate axons and myelin sheath formation as our previous article [3]. Eight weeks after surgery, rats were anesthetized and perfused transcardially with a phosphate-buffered 2% paraformaldehyde-2.5% glutaraldehyde fixative. The tissue sections (1 µm) were stained with toluidine blue. Neuropathological examination was conducted using light microscopy. The quantitative analysis of the injured nerve fiber and nerve fibers with degeneration and demyelination was performed. Then, the g-ratio (axon width/fiber width) was presented using three-dimensional SigmaPlot analysis.

**5. *In vitro* HucMSC-EX release assay**

The *in vitro* exosome release assay was modified according to our previous study [4]. Gelfoam sponges (2 x 2 x 2 mm^3^) were placed into 2 mg/ml exosome solution and centrifuged at 3000g for 10 min at 4 °C to remove bubbles in Gelfoam. Following overnight incubation at 37 °C, HucMSC-EX-loaded Gelfoam were transferred into a 96-well cell culture plate with 200 µl of sterilized saline in each well at 37 °C. Supernatants were then retrieved on day 1, 2, 3, 4, 5, 6 and 7. Gelfoam alone was used as a negative control. Next, analysis of the total protein concentration in each sample was performed using BCA Protein Assay Kit. The total exosome protein level in each sample was normalized by deducting the negative control.

**6. *In vitro* HucMSC-EX uptake assay**

PC12 cells were divided into three groups: Group 1 is PC12 cell alone (PC12), Group 2 is PC12 cells with unlabeled exosomes (PC12 + EX, unlabeled), and Group 3 is PC12 cells with green fluorescence-labeled exosomes (PC12 + EX, label). The exosomes (1 × 10^11^ particles/ml, 1 mg/ml, 100 μg) were stained by ExoGlowTM-Protein EV Labeling kit (green, System Biosciences, SBI). PC12 cells (1 × 10^5^ cells/ml) were seeded into 12-well cell culture plate with complete culture medium containing 10% horse serum and 5% FBS. Next, exosomes with or without green fluorescence (Group 2 and 3) were added into the cell cultures and incubated for 12 hours. Same volume of culture medium was used in control group (Group 1). Following cell suspension and 4% paraformaldehyde fixation, the nuclei were stained by DAPI, and the fluorescence images were collected by Olympus FV300 confocal laser scanning microscope.

**Supplementary reference:**

[1] X. Liu, X. Wang, J. Lu, Tenuifoliside A promotes neurite outgrowth in PC12 cells via the PI3K/AKT and MEK/ERK/CREB signaling pathways, Mol Med Rep 12(5) (2015) 7637-7642.

[2] K. Pemberton, B. Mersman, F. Xu, Using ImageJ to Assess Neurite Outgrowth in Mammalian Cell Cultures: Research Data Quantification Exercises in Undergraduate Neuroscience Lab, Journal of undergraduate neuroscience education : JUNE : a publication of FUN, Faculty for Undergraduate Neuroscience 16(2) (2018) A186-a194.

[3] L.C. Chu, M.L. Tsaur, C.S. Lin, Y.C. Hung, T.Y. Wang, C.C. Chen, J.K. Cheng, Chronic intrathecal infusion of gabapentin prevents nerve ligation-induced pain in rats, BJA: British Journal of Anaesthesia 106(5) (2011) 699-705.

[4] J.M. Hsu, S.J. Shiue, K.D. Yang, H.S. Shiue, Y.W. Hung, P. Pannuru, R. Poongodi, H.Y. Lin, J.K. Cheng, Locally Applied Stem Cell Exosome-Scaffold Attenuates Nerve Injury-Induced Pain in Rats, Journal of pain research 13 (2020) 3257-3268.
