# Supplementary figures and images for: Stem cell exosome-loaded Gelfoam improves locomotor dysfunction and neuropathic pain in a rat model of spinal cord injury
Source: Stem Cell Res Ther. 2024 May 20;15:143. doi: 10.1186/s13287-024-03758-5 (PMC11103960; doi:10.1186/s13287-024-03758-5)

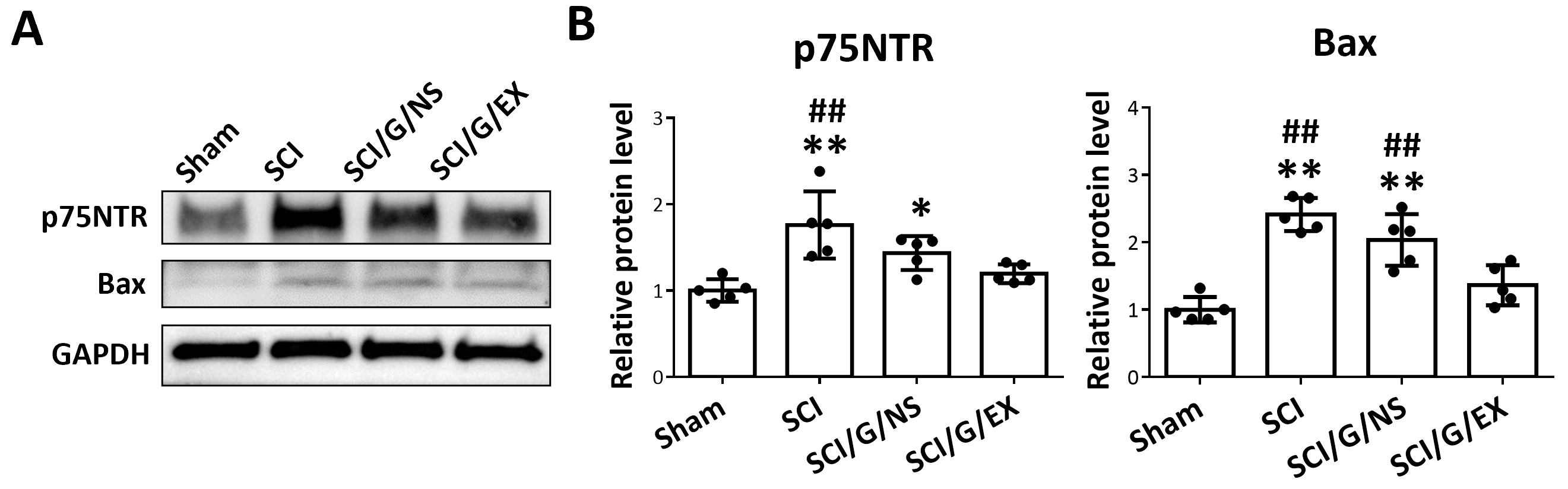

Supplement: Supplementary file 2 — Additional file 2: fig. S1.. (A) Graphical illustration of hemisection surgery at T9 spinal cord on the right side and implantation of exosome-loaded Gelfoam in the lesion site. (B)In vitro exosome release assay demonstrating the percentage of soaked exosomes released from the Gelfoam from day 1 to 7 in the 96-well plate. Data are presented as mean ± standard deviation (n = 3). (C) Confocal microscopy images and orthogonal views (Z-stack projection) of internalized Exo-fluorescent green-labeled HucMSC-EX by F4/80+ macrophages around the lesion site on day 3 post-surgery. The yellow spots indicate exosome uptake. Scale bar: 25 μm as indicated. HucMSC-EX: human umbilical cord mesenchymal stem cell-derived exosome; SCI: spinal cord injury; EX: exosome. [file 13287_2024_3758_MOESM2_ESM.tif]

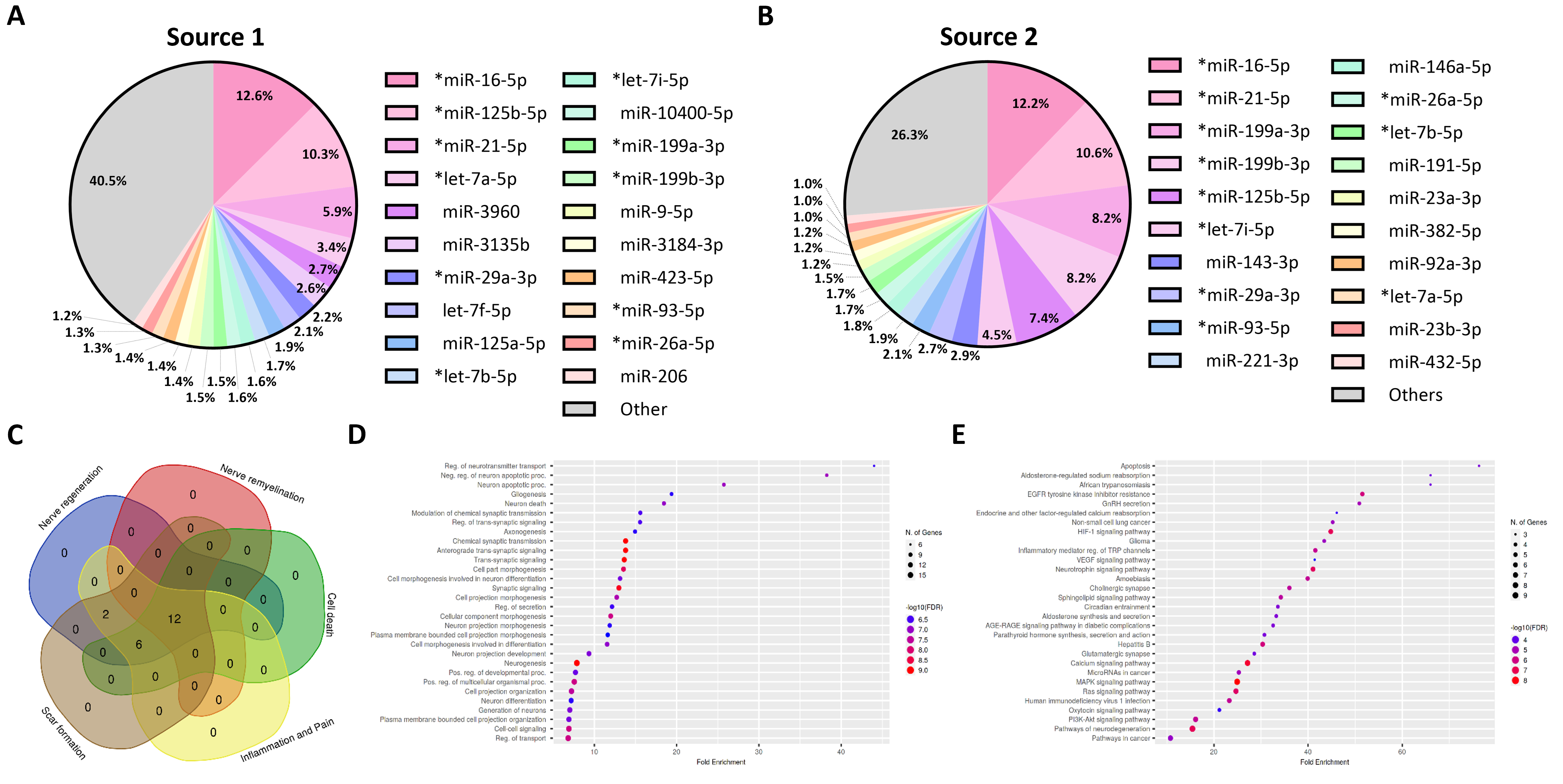

Supplement: Supplementary file 3 — Additional file 3: fig. S1. HucMSC-EX treatment decreased spinal cord injury-induced axon degeneration around the lesion site at week 8 after surgery. (A) Representative histological images of toluidine blue staining around the T9 lesion site. Scale bar: 40 μm as indicated. (B) The axon width, fiber width, and g-ratio of four groups. The high-resolution image (100) was used to calculate the g-ratio (ratio of axon width to fiber width). Data are presented as mean ± standard deviation. *p < 0.05, **p < 0.01 compared with the sham group, ##p < 0.01 compared with the SCI/G/EX group, and $p < 0.05 compared with the SCI/G/NS group through one-way ANOVA with post hoc Tukey’s multiple comparisons test. (C) The correlations between axon width, fiber width, and g-ratio are demonstrated. HucMSC-EX: human umbilical cord mesenchymal stem cell-derived exosome; SCI: spinal cord injury; G: Gelfoam; NS: normal saline. [file 13287_2024_3758_MOESM3_ESM.tif]

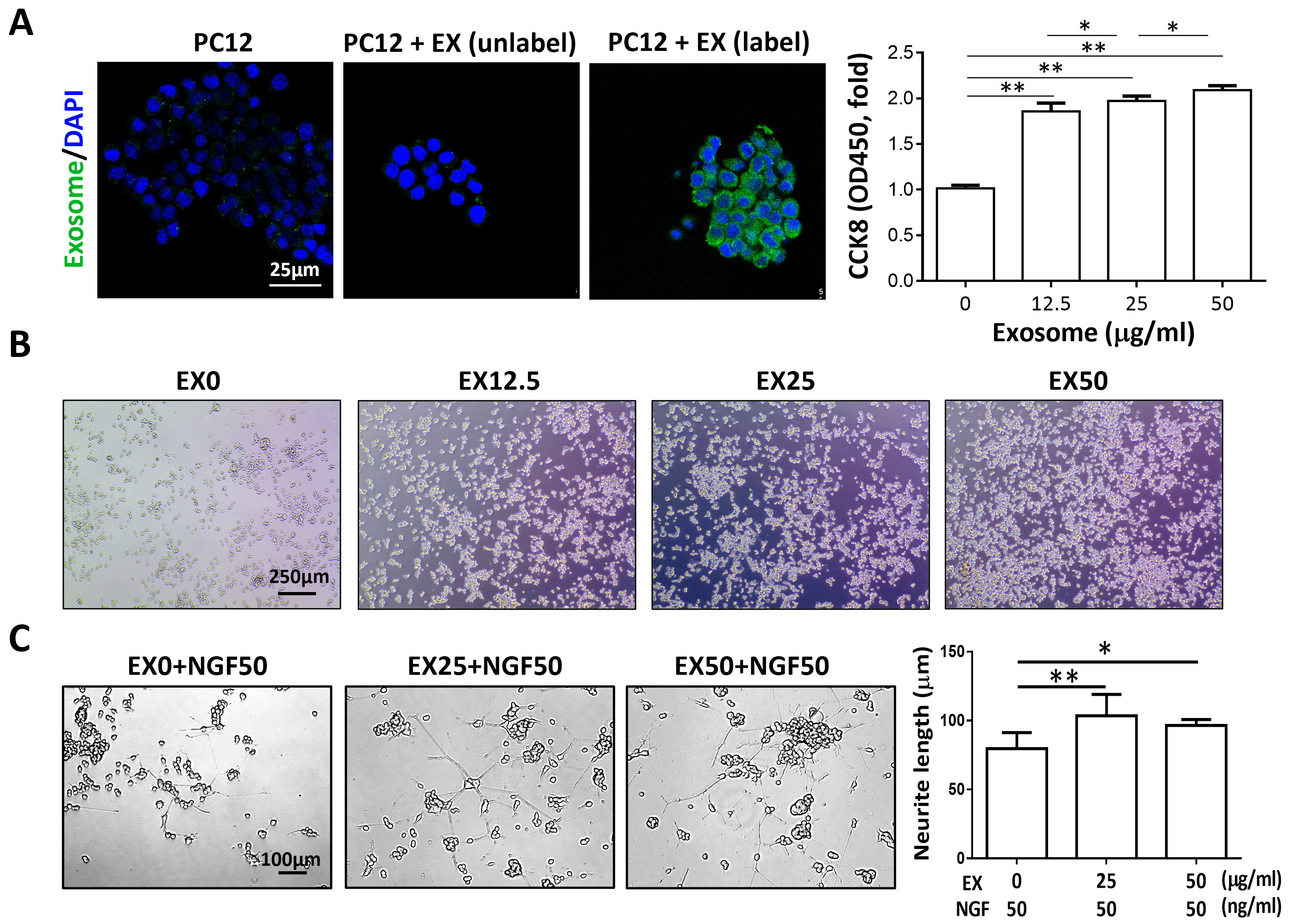

Supplement: Supplementary file 4 — Additional file 4: fig. S1. HucMSC-EX treatment improved the destruction of motor neurons around the lesion site at week 8 after spinal cord injury. (A, B) Representative immunofluorescence images and relative integrated intensities of choline acetyltransferase (ChAT, motor neuron marker, green) and neuronal nuclei (NeuN, red) around the T9 lesion site in four groups. The white box shows the magnification of the specific area. Scale bar: 100, 50, and 25 μm as indicated. n = 3 in each group. Data are presented as mean ± standard deviation, taking the Sham group as 100%. *p < 0.05 and **p < 0.01 compared with the sham group through one-way ANOVA with post hoc Tukey’s multiple comparisons test, n = 3 in each group. HucMSC-EX: human umbilical cord mesenchymal stem cell-derived exosome; SCI: spinal cord injury; G: Gelfoam; NS; normal saline; Ipsi: ipsilateral; Contra: contralateral. [file 13287_2024_3758_MOESM4_ESM.tif]

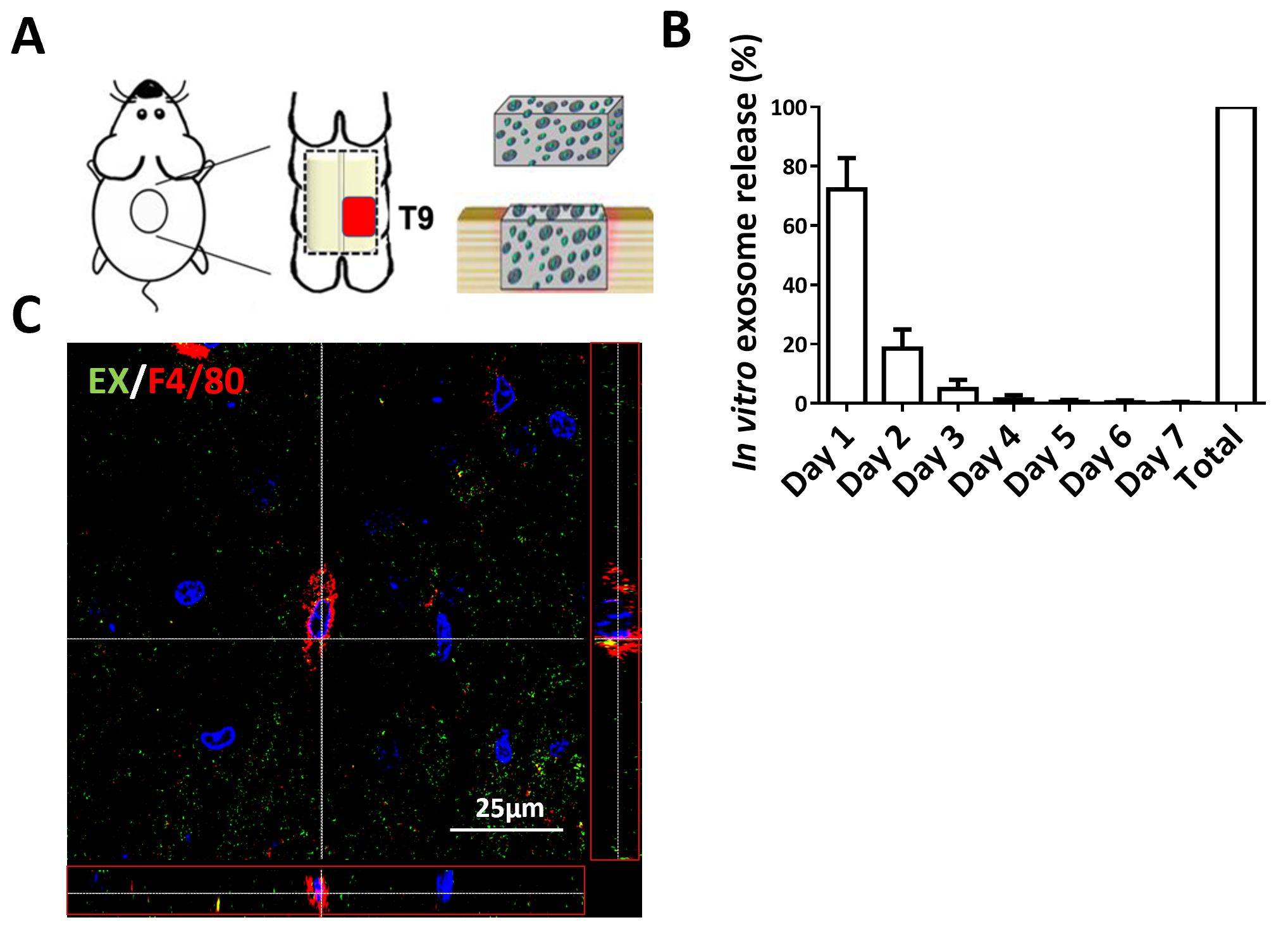

Supplement: Supplementary file 5 — Additional file 5: fig. S1. HucMSC-EX-loaded Gelfoam implantation attenuated spinal cord injury-induced apoptosis at week 8 after surgery. (A, B) Representative Western blots and relative protein levels of p75NTR and Bax around the T9 lesion site at week 8 after spinal cord injury. GAPDH was used as an internal control. Data are presented as mean ± standard deviation, taking the Sham group as 100%. *p < 0.05, **p < 0.01 compared with the sham group, and ##p < 0.01 compared with the SCI/G/EX group by one-way ANOVA with post hoc Tukey’s multiple comparisons test, n = 5 in each group. HucMSC-EX: human umbilical cord mesenchymal stem cell-derived exosome; SCI: spinal cord injury; G: Gelfoam; NS: normal saline. [file 13287_2024_3758_MOESM5_ESM.tif]

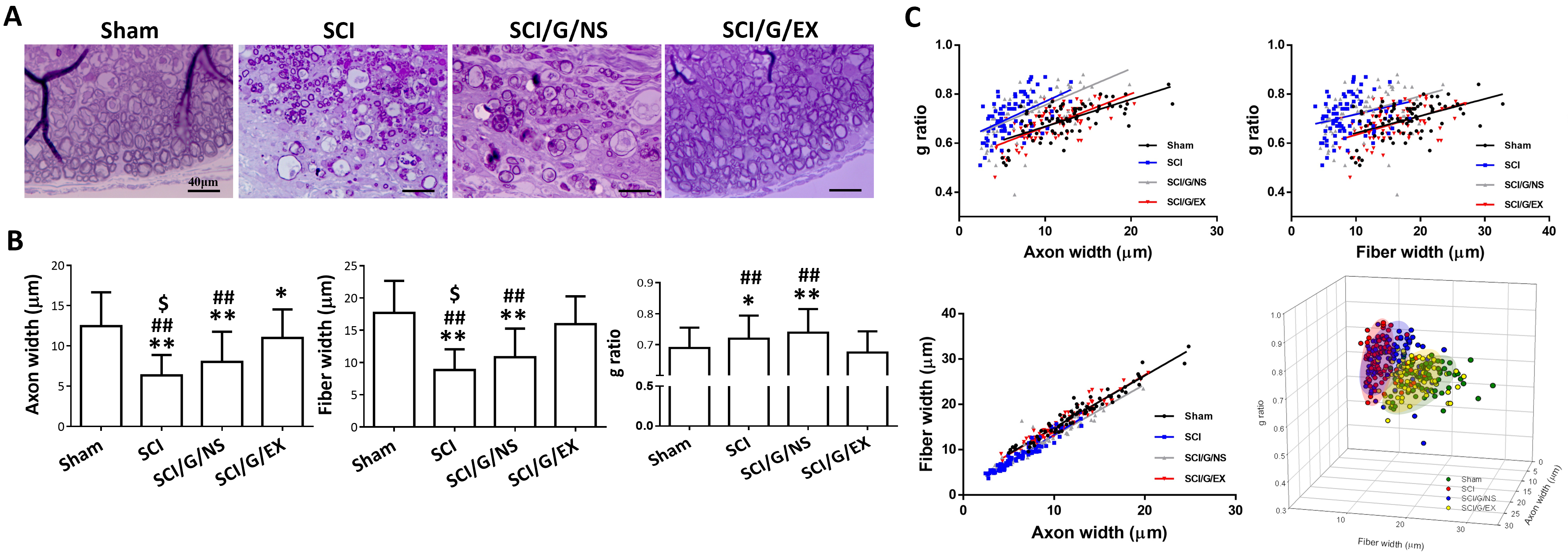

Supplement: Supplementary file 6 — Additional file 6: fig. S1. Identification of miRNA content of human umbilical cord mesenchymal stem cell-derived exosome (HucMSC-EX) through next-generation sequencing (NGS). (A, B) The top 20 abundant miRNAs of HucMSC-EX from two sources. (C) Distribution of the abundant top 20 miRNA in five relevant biological functional domains (nerve regeneration, nerve remyelination, glial scar formation, cell death, and inflammation/pain) through the Venn diagram. (D, E) The hot map of the biological process and Kyoto Encyclopedia of Genes and Genomes (KEGG) pathway analysis in the top 20 abundant miRNAs using ShinyGo (http://bioinformatics.sdstate.edu/go/). [file 13287_2024_3758_MOESM6_ESM.tif]

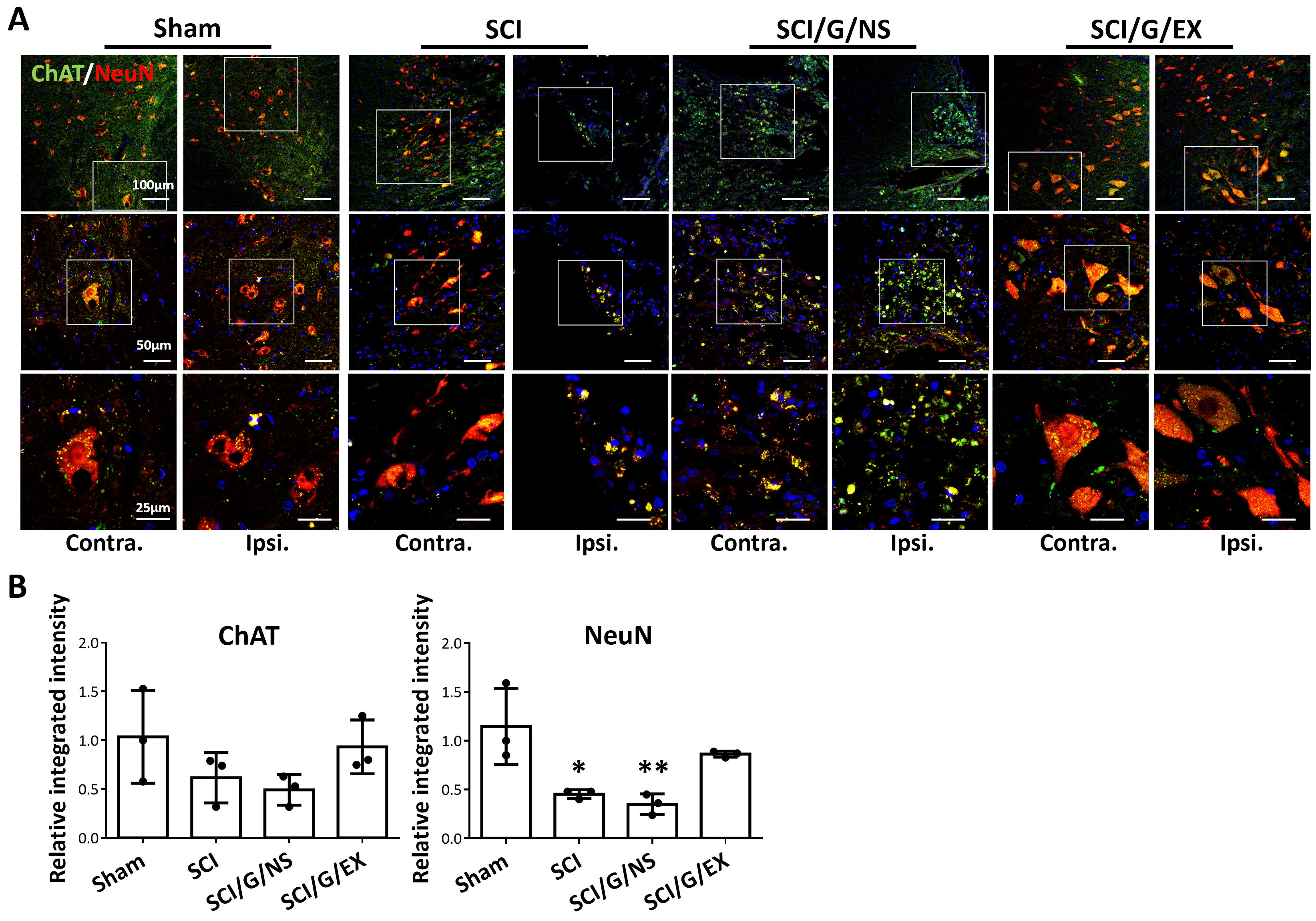

Supplement: Supplementary file 10 — Additional file 7: fig. S1. HucMSC-EX can promote cell proliferation and neurite outgrowth in PC12 cells. (A)In vitro exosome uptake assay. PC12 cells were divided into three groups: Group 1 is PC12 cells alone, Group 2 is PC12 cells with unlabeled exosomes, and Group 3 is PC12 cells with Exo-Green-labeled exosomes. (B) Effects of exosome on cell proliferation in PC12 cells. PC12 cells were treated with HucMSC-EX (12.5, 25, and 50 µg/ml) for 72 h. Cell Counting Kit-8 (CCK-8) was used to evaluate the effects of exosomes on PC12 cell proliferation. (C) Effects of exosome on neurite outgrowth in NGF-treated PC12 cells. PC12 cells were pretreated with HucMSC-EX (25 and 50 µg/ml) for 24 h and then stimulated by NGF (50 ng/ml) for another 5 days. *p < 0.05 and **p < 0.05. HucMSC-EX: human umbilical cord mesenchymal stem cell-derived exosome; NGF: neural growth factor. [file 13287_2024_3758_MOESM10_ESM.tif]
