# Supplementary material for: Stem cell exosome-loaded Gelfoam improves locomotor dysfunction and neuropathic pain in a rat model of spinal cord injury
Source: Stem Cell Res Ther. 2024 May 20;15:143. doi: 10.1186/s13287-024-03758-5 (PMC11103960; doi:10.1186/s13287-024-03758-5)
Supplement: Supplementary file 7 — Additional file 6: table S1. The top 20 abundant miRNAs of source 1 HucMSC-EX with their predicted target genes (retrieved from TargetScan and miRanda miRNA database) are categorized into five relevant biological functional domains: nerve regeneration, remyelination, glial scar formation, cell death, and inflammation/pain. [file 13287_2024_3758_MOESM7_ESM.docx]

**Additional file 6: Table S1.** The top 20 abundant miRNAs of source 1 HucMSC-EX with their predicted target genes (retrieved from TargetScan and miRanda miRNA database) were categorized into five relevant biological functional domains: nerve regeneration, remyelination, glial scar formation, cell death, and inflammation/pain.

| **Nerve regeneration** | |
| --- | --- |
| NEFH  (NF200) | miR-21-5p, miR-3960, miR-3135b, miR-29a-3p, miR-10400-5p, miR-9-5p, miR-93-5p |
| DLG4  (PSD95) | miR-125b-5p, miR-3960, miR-3135b, miR-125a-5p, let-7i-5p, miR-10400-5p, miR-423-5p, miR-26a-5p, miR-206 |
| SYP  (Synaptophysin) | miR-16-5p, miR-125b-5p, miR-3135b, miR-29a-3p, miR-125a-5p, miR-423-5p, miR-3184-3p, miR-93-5p |
| GAP43 | miR-16-5p, miR-3960, miR-199a-3p, miR-199b-3p, miR-9-5p, miR-26a-5p |
| NTRK1  (TrkA) | miR-16-5p, miR-3960, miR-3135b, miR-29a-3p, let-7b-5p, miR-10400-5p, miR-93-5p |
| GDNF | miR-16-5p, miR-125b-5p, miR-3960, miR-3135b, miR-125a-5p, miR-29a-3p, let-7f-5p, miR-10400-5p, miR-9-5p, miR-423-5p, miR-93-5p |
| Rbfox3  (NeuN) | miR-125b-5p, miR-3960, miR-3135b, miR-125a-5p, miR-29a-3p, miR-423-5p, miR-3184-3p |
| CHAT | miR-16-5p, miR-21-5p, miR-3960, miR-3135b, miR-10400-5p, miR-3184-3p, miR-93-5p |
| **Nerve** **remyelination** (oligodendrocytes, myelin) | |
| OLIG2 | miR-16-5p, miR-3960, miR-10400-5p, miR-423-5p, miR-93-5p, miR-206 |
| MBP | miR-16-5p, miR-125b-5p, miR-21-5p, miR-3960, miR-3135b, let-7f-5p, miR-125a-5p, miR-10400-5p, miR-9-5p, miR-423-5p, miR-93-5p |
| **Glial scar formation** | |
| GFAP | miR-16-5p, miR-125b-5p, miR-3960, miR-3135b, miR-125a-5p, miR-10400-5p, miR-199a-3p, miR-199b-3p, miR-9-5p, miR-93-5p, miR-423-5p, miR-206 |
| VIM  (vimentin) | miR-21-5p, miR-3960, miR-3135b, miR-10400-5p, miR-423-5p, miR-3184-3p |
| CSPG4  (NG2) | miR-16-5p, miR-125b-5p, let-7a-5p, miR-3960, miR-3135b, miR-29a-3p, let-7f-5p, miR-125a-5p, let-7b-5p, let-7i-5p, miR-10400-5p, miR-423-5p, miR-3184-3p, miR-93-5p, miR-26a-5p, miR-206 |
| CSPG5 | miR-16-5p, miR-3960, miR-3135b, let-7f-5p, miR-10400-5p, miR-199a-3p, miR-199b-3p, miR-9-5p, miR-423-5p, miR-26a-5p |
| **Cell death** | |
| NGFR  (p75NTR) | miR-16-5p, let-7a-5p, miR-3960, miR-3135b, miR-125a-5p, let-7b-5p, miR-10400-5p, miR-423-5p, miR-206 |
| Bax | let-7a-5p, miR-29a-3p, let-7f-5p, let-7b-5p, let-7i-5p, miR-3184-3p |
| Bcl-2 | miR-16-5p, miR-125b-5p, miR-21-5p, let-7a-5p, miR-3960, miR-3135b, miR-29a-3p, miR-125a-5p, let-7b-5p, miR-10400-5p, miR-9-5p, miR-423-5p, miR-93-5p, miR-26a-5p, miR-206 |
| **Inflammation/Pain** | |
| VEGF(A) | miR-16-5p, miR-125b-5p, miR-21-5p, let-7a-5p, miR-3135b, miR-29a-3p, let-7f-5p, miR-125a-5p, let-7b-5p, let-7i-5p, miR-9-5p, miR-423-5p, miR-3184-3p, miR-93-5p, miR-26a-5p, miR-206 |
| NOS2  (iNOS) | miR-16-5p, let-7a-5p, miR-3960, miR-3135b, miR-29a-3p, let-7f-5p, let-7b-5p, let-7i-5p, miR-10400-5p, miR-423-5p, miR-3184-3p, miR-93-5p, miR-26a-5p, miR-206 |
| AIF1  (Iba-1) | miR-3135b, let-7i-5p, miR-423-5p |
| TLR4 | miR-16-5p, let-7a-5p, miR-21-5p, miR-3135b, miR-29a-3p, let-7f-5p, let-7b-5p, let-7i-5p, miR-199a-3p, miR-199b-3p, miR-3184-3p, miR-93-5p, miR-26a-5p, miR-206 |
| BDNF | miR-16-5p, miR-21-5p, let-7a-5p, miR-3960, miR-3135b, miR-29a-3p, let-7f-5p, let-7b-5p, let-7i-5p, miR-10400-5p, miR-199a-3p, miR-199b-3p, miR-9-5p, miR-423-5p, miR-3184-3p, miR-206 |
| NTRK2  (TrkB) | miR-16-5p, miR-125b-5p, miR-21-5p, let-7a-5p, miR-3960, miR-3135b, miR-29a-3p, let-7f-5p, miR-125a-5p, let-7i-5p, miR-10400-5p, miR-199a-3p, miR-199b-3p, miR-9-5p, miR-423-5p, miR-3184-3p, miR-93-5p, miR-26a-5p, miR-206 |
| TRPV1 | miR-16-5p, let-7a-5p, miR-3960, miR-3135b, miR-29a-3p, let-7f-5p, let-7b-5p, let-7i-5p, miR-10400-5p, miR-9-5p, miR-423-5p |
| CACNA1H (Cav3.2) | miR-16-5p, miR-125b-5p, let-7a-5p, miR-3960, miR-3135b, miR-29a-3p, let-7f-5p, miR-125a-5p, let-7b-5p, let-7i-5p, miR-10400-5p, miR-199a-3p, miR-199b-3p, miR-423-5p, miR-3184-3p, miR-93-5p |
| CACNA2D1  (alpha-2/delta-1) | miR-16-5p, let-7a-5p, miR-3135b, miR-29a-3p, let-7f-5p, let-7b-5p, let-7i-5p, miR-199a-3p, miR-199b-3p, miR-9-5p, miR-423-5p, miR-26a-5p, miR-206 |
| PRKCG  (PKC-γ) | miR-125b-5p, miR-21-5p, miR-3960, miR-3135b, miR-125a-5p, miR-10400-5p, miR-423-5p, miR-3184-3p |
| MAPK3  (Erk1/2) | miR-16-5p, miR-125b-5p, let-7a-5p, miR-3960, miR-3135b, let-7f-5p, miR-125a-5p, let-7b-5p, let-7i-5p, miR-423-5p, miR-206 |

HucMSC-EX: human umbilical cord mesenchymal stem cell-derived exosome. Retrieved from two databanks (TargetScan and miRanda).
