# Supplementary material for: Stem cell exosome-loaded Gelfoam improves locomotor dysfunction and neuropathic pain in a rat model of spinal cord injury
Source: Stem Cell Res Ther. 2024 May 20;15:143. doi: 10.1186/s13287-024-03758-5 (PMC11103960; doi:10.1186/s13287-024-03758-5)
Supplement: Supplementary file 8 — Additional file 6: table S2. References to support the potential therapeutic effects of the top 20 abundant miRNAs of source 1 and source 2 HucMSC-EX in spinal cord injury and neuropathic pain. [file 13287_2024_3758_MOESM8_ESM.docx]

**Additional file 6: Table S2.** References to support the potential therapeutic effects of the top 20 abundant miRNAs of source 1 and source 2 HucMSC-EX in SCI and NP.

| Source 1 | | | Source 2 | | |
| --- | --- | --- | --- | --- | --- |
| No. | **miRNA** | **Reference (SCI/NP)** | **No.** | **miRNA** | **Reference (SCI/NP)** |
| 1 | *miR-16-5p | [1]/[2-4] | **1** | *miR-16-5p | [1]/[2-4] |
| 2 | *miR-125b-5p | [5, 6]/[7, 8] | **2** | *miR-21-5p | [9-17]/[18, 19] |
| 3 | *miR-21-5p | [9-17]/[18, 19] | **3** | *miR-199a-3p | [12, 20]/none |
| 4 | *let-7a-5p | [21]/[4] | **4** | *miR-199b-3p | none/none |
| 5 | miR-3960 | none/none | **5** | *miR-125b-5p | [5, 6]/[7, 8] |
| 6 | miR-3135b | none/none | **6** | *let-7i-5p | none/none |
| 7 | *miR-29a-3p | [22]/none | **7** | miR-143-3p | [23, 24]/[25] |
| 8 | let-7f-5p | none/none | **8** | *miR-29a-3p | [22]/none |
| 9 | miR-125a-5p | none/[8, 26] | **9** | *miR-93-5p | none/[27] |
| 10 | *let-7b-5p | none/none | **10** | miR-221-3p | [13]/none |
| 11 | *let-7i-5p | none/none | **11** | miR-146a-5p | [6, 28-30]/[31-34] |
| 12 | miR-10400-5p | none/none | **12** | *miR-26a-5p | none/[35, 36] |
| 13 | *miR-199a-3p | [12, 20]/none | **13** | *let-7b-5p | none/none |
| 14 | *miR-199b-3p | none/none | **14** | miR-191-5p | [6]/none |
| 15 | miR-9-5p | [37, 38]/none | **15** | miR-23a-3p | [39, 40]/[41] |
| 16 | miR-3184-3p | none/none | **16** | miR-382-5p | none/none |
| 17 | miR-423-5p | [42, 43]/[44] | **17** | miR-92a-3p | [45]/none |
| 18 | *miR-93-5p | none/[27] | **18** | *let-7a-5p | [21]/[4] |
| 19 | *miR-26a-5p | none/[35, 36] | **19** | miR-23b-3p | none/none |
| 20 | miR-206 | [46]/[47-51] | **20** | miR-432-5p | none/none |

HucMSC-EX: human umbilical cord mesenchymal stem cell-derived exosome; SCI: spinal cord injury; NP: neuropathic pain. *Overlapping miRNAs between source 1 and source 2 HucMSC-EX.

**Reference:**

1. Wang, N., et al., *Integrated analysis of competing endogenous RNA (ceRNA) networks in subacute stage of spinal cord injury.* Gene, 2020. **726**: p. 144171.

2. Cai, G., et al., *Network Analysis of miRNA and mRNA Changes in the Prelimbic Cortex of Rats With Chronic Neuropathic Pain: Pointing to Inflammation.* Front Genet, 2020. **11**: p. 612.

3. Tang, S., et al., *Identification of key candidate genes in neuropathic pain by integrated bioinformatic analysis.* J Cell Biochem, 2020. **121**(2): p. 1635-1648.

4. Zhang, D., et al., *Intradermal miR-16-5p targets Akt3 and reduces RTX-induced postherpetic neuralgia-mimic pain in mice.* Eur J Pharmacol, 2023. **946**: p. 175665.

5. Ding, S.Q., et al., *Identification of serum exosomal microRNAs in acute spinal cord injured rats.* Exp Biol Med (Maywood), 2019. **244**(14): p. 1149-1161.

6. Paim, L.R., et al., *Circulating microRNAs, Vascular Risk, and Physical Activity in Spinal Cord-Injured Subjects.* J Neurotrauma, 2019. **36**(6): p. 845-852.

7. Bali, K.K., et al., *Sources of individual variability: miRNAs that predispose to neuropathic pain identified using genome-wide sequencing.* Mol Pain, 2014. **10**: p. 22.

8. Chen, P., et al., *Identification of Slc6a19os and SOX11 as Two Novel Essential Genes in Neuropathic Pain Using Integrated Bioinformatic Analysis and Experimental Verification.* Front Neurosci, 2021. **15**: p. 627945.

9. Lv, X., J. Liang, and Z. Wang, *MiR-21-5p reduces apoptosis and inflammation in rats with spinal cord injury through PI3K/AKT pathway.* Panminerva Med, 2020.

10. Wang, J., et al., *The role of lncRNA-MEG/miR-21-5p/PDCD4 axis in spinal cord injury.* Am J Transl Res, 2021. **13**(2): p. 646-658.

11. Zhou, X., et al., *Mesenchymal stem cell derived EVs mediate neuroprotection after spinal cord injury in rats via the microRNA-21-5p/FasL gene axis.* Biomed Pharmacother, 2019. **115**: p. 108818.

12. Chen, F., J. Han, and D. Wang, *Identification of key microRNAs and the underlying molecular mechanism in spinal cord ischemia-reperfusion injury in rats.* PeerJ, 2021. **9**: p. e11454.

13. Wang, D., et al., *Sevoflurane pretreatment regulates abnormal expression of MicroRNAs associated with spinal cord ischemia/reperfusion injury in rats.* Ann Transl Med, 2021. **9**(9): p. 752.

14. Wang, Y., H. Yi, and Y. Song, *miRNA Therapy in Laboratory Models of Acute Spinal Cord Injury in Rodents: A Meta-analysis.* Cell Mol Neurobiol, 2023. **43**(3): p. 1147-1161.

15. Shang, Z., et al., *MicroRNA expression profile in the spinal cord injured rat neurogenic bladder by next-generation sequencing.* Transl Androl Urol, 2020. **9**(4): p. 1585-1602.

16. Tang, Y., et al., *Time-specific microRNA changes during spinal motoneuron degeneration in adult rats following unilateral brachial plexus root avulsion: ipsilateral vs. contralateral changes.* BMC Neurosci, 2014. **15**: p. 92.

17. Wang, W., et al., *[Identification of potential traumatic spinal cord injury related circular RNA-microRNA networks by sequence analysis].* Zhongguo Xiu Fu Chong Jian Wai Ke Za Zhi, 2020. **34**(2): p. 213-219.

18. Zhong, L., et al., *miR-21-5p inhibits neuropathic pain development via directly targeting C-C motif ligand 1 and tissue inhibitor of metalloproteinase-3.* J Cell Biochem, 2019. **120**(10): p. 16614-16623.

19. Leinders, M., et al., *Aberrant microRNA expression in patients with painful peripheral neuropathies.* J Neurol Sci, 2017. **380**: p. 242-249.

20. Wang, Y., et al., *Umbilical mesenchymal stem cell-derived exosomes facilitate spinal cord functional recovery through the miR-199a-3p/145-5p-mediated NGF/TrkA signaling pathway in rats.* Stem Cell Res Ther, 2021. **12**(1): p. 117.

21. Wang, Y., et al., *Micro-RNA let-7a-5p Derived From Mesenchymal Stem Cell-Derived Extracellular Vesicles Promotes the Regrowth of Neurons in Spinal-Cord-Injured Rats by Targeting the HMGA2/SMAD2 Axis.* Front Mol Neurosci, 2022. **15**: p. 850364.

22. Wang, X., et al., *Hypoxia-treated umbilical mesenchymal stem cell alleviates spinal cord ischemia-reperfusion injury in SCI by circular RNA circOXNAD1/ miR-29a-3p/ FOXO3a axis.* Biochem Biophys Rep, 2023. **34**: p. 101458.

23. Hu, Y., et al., *MicroRNA expression profile of human umbilical vein endothelial cells in response to coxsackievirus A10 infection reveals a potential role of miR-143-3p in maintaining the integrity of the blood-brain barrier.* Front Cell Infect Microbiol, 2023. **13**: p. 1217984.

24. Yang, Y., et al., *Differential Expression of microRNAs and Target Genes Analysis in Olfactory Ensheathing Cell-derived Extracellular Vesicles Versus Olfactory Ensheathing Cells.* Curr Stem Cell Res Ther, 2024. **19**(1): p. 116-125.

25. Norcini, M., et al., *Intrathecal Injection of miR-133b-3p or miR-143-3p Prevents the Development of Persistent Cold and Mechanical Allodynia Following a Peripheral Nerve Injury in Rats.* Neuroscience, 2018. **386**: p. 223-239.

26. Kasimu, A., et al., *miR-125a-5p in astrocytes attenuates peripheral neuropathy in type 2 diabetic mice through targeting TRAF6.* Endocrinol Diabetes Nutr (Engl Ed), 2022. **69**(1): p. 43-51.

27. Staal, R., et al., *P2X7 receptor-mediated release of microglial prostanoids and miRNAs correlates with reversal of neuropathic hypersensitivity in rats.* Eur J Pain, 2022. **26**(6): p. 1304-1321.

28. Lai, X., et al., *miR-146a-5p-modified hUCMSC-derived exosomes facilitate spinal cord function recovery by targeting neurotoxic astrocytes.* Stem Cell Res Ther, 2022. **13**(1): p. 487.

29. Ni, S., et al., *EZH2 Mediates miR-146a-5p/HIF-1alpha to Alleviate Inflammation and Glycolysis after Acute Spinal Cord Injury.* Mediators Inflamm, 2021. **2021**: p. 5591582.

30. He, Y., et al., *Zhenbao pill protects against acute spinal cord injury via miR-146a-5p regulating the expression of GPR17.* Biosci Rep, 2018. **38**(1).

31. Weng, H.R., et al., *EZH2 Methyltransferase Regulates Neuroinflammation and Neuropathic Pain.* Cells, 2023. **12**(7).

32. Lu, Y., et al., *MicroRNA-146a-5p attenuates neuropathic pain via suppressing TRAF6 signaling in the spinal cord.* Brain Behav Immun, 2015. **49**: p. 119-29.

33. Zhang, Q., D. Zhu, and Q. Li, *LncRNA CRNDE exacerbates neuropathic pain in chronic constriction injury-induced(CCI) rats through regulating miR-146a-5p/WNT5A pathway.* Bioengineered, 2021. **12**(1): p. 7348-7359.

34. Pham, T.L., et al., *miRNA 146a-5p-loaded poly(d,l-lactic-co-glycolic acid) nanoparticles impair pain behaviors by inhibiting multiple inflammatory pathways in microglia.* Nanomedicine (Lond), 2020. **15**(11): p. 1113-1126.

35. Lu, Y., et al., *Human PMSCs-derived small extracellular vesicles alleviate neuropathic pain through miR-26a-5p/Wnt5a in SNI mice model.* J Neuroinflammation, 2022. **19**(1): p. 221.

36. Zhang, Y., et al., *Effects of miR-26a-5p on neuropathic pain development by targeting MAPK6 in in CCI rat models.* Biomed Pharmacother, 2018. **107**: p. 644-649.

37. He, X., et al., *Exosomal miR-9-5p derived from BMSCs alleviates apoptosis, inflammation and endoplasmic reticulum stress in spinal cord injury by regulating the HDAC5/FGF2 axis.* Mol Immunol, 2022. **145**: p. 97-108.

38. Wang, F., et al., *Lithium alleviated spinal cord injury (SCI)-induced apoptosis and inflammation in rats via BDNF-AS/miR-9-5p axis.* Cell Tissue Res, 2021. **384**(2): p. 301-312.

39. Wu, C., et al., *Exosome miR-23a-3p from Osteoblast Alleviates Spinal Cord Ischemia/Reperfusion Injury by Down-Regulating KLF3-Activated CCNL2 Transcription.* Dev Neurosci, 2022. **44**(3): p. 121-130.

40. Peng, P., et al., *Exosomes-mediated phenotypic switch of macrophages in the immune microenvironment after spinal cord injury.* Biomed Pharmacother, 2021. **144**: p. 112311.

41. Ghafouri-Fard, S., et al., *A review on the role of LINC00472 in malignant and non-malignant disorders.* Pathol Res Pract, 2023. **247**: p. 154549.

42. Cheng, J., et al., *Ameliorative effects of miR-423-5p against polarization of microglial cells of the M1 phenotype by targeting a NLRP3 inflammasome signaling pathway.* Int Immunopharmacol, 2021. **99**: p. 108006.

43. Wu, R., et al., *Differential Circular RNA Expression Profiles Following Spinal Cord Injury in Rats: A Temporal and Experimental Analysis.* Front Neurosci, 2019. **13**: p. 1303.

44. Pan, X., et al., *Loss of SNHG4 Attenuated Spinal Nerve Ligation-Triggered Neuropathic Pain through Sponging miR-423-5p.* Mediators Inflamm, 2020. **2020**: p. 2094948.

45. He, S., et al., *MicroRNA-92a-3p enhances functional recovery and suppresses apoptosis after spinal cord injury via targeting phosphatase and tensin homolog.* Biosci Rep, 2020. **40**(5).

46. Chakrabarti, M., N.L. Banik, and S.K. Ray, *MiR-7-1 potentiated estrogen receptor agonists for functional neuroprotection in VSC4.1 motoneurons.* Neuroscience, 2014. **256**: p. 322-33.

47. Chen, Z.L., et al., *Suppression of MALAT1 ameliorates chronic constriction injury-induced neuropathic pain in rats via modulating miR-206 and ZEB2.* J Cell Physiol, 2019. **234**(9): p. 15647-15653.

48. Sun, W., L. Zhang, and R. Li, *Overexpression of miR-206 ameliorates chronic constriction injury-induced neuropathic pain in rats via the MEK/ERK pathway by targeting brain-derived neurotrophic factor.* Neurosci Lett, 2017. **646**: p. 68-74.

49. Guo, J.B., et al., *Network and pathway-based analysis of microRNA role in neuropathic pain in rat models.* J Cell Mol Med, 2019. **23**(7): p. 4534-4544.

50. Xu, Y., et al., *Circulating microRNA expression profile: a novel potential predictor for chronic nervous lesions.* Acta Biochim Biophys Sin (Shanghai), 2014. **46**(11): p. 942-9.

51. Kusuda, R., et al., *Differential expression of microRNAs in mouse pain models.* Mol Pain, 2011. **7**: p. 17.
