# Supplementary material for: Stem cell exosome-loaded Gelfoam improves locomotor dysfunction and neuropathic pain in a rat model of spinal cord injury
Source: Stem Cell Res Ther. 2024 May 20;15:143. doi: 10.1186/s13287-024-03758-5 (PMC11103960; doi:10.1186/s13287-024-03758-5)
Supplement: Supplementary file 9 — Additional file 6: table S3. The exosome-downregulated proteins in this study and their possible related top 20 abundant miRNAs in source 1 and source 2 HucMSC-EX. [file 13287_2024_3758_MOESM9_ESM.docx]

**Additional file 6: Table S3.** The exosome-downregulated proteins in this study and their possible related top 20 abundant miRNAs in source 1 and source 2 HucMSC-EX

| Protein | miRNA (source 1) | miRNA (source 2) |
| --- | --- | --- |
| GFAP | *miR-16-5p, *miR-125b-5p, miR-3960, miR-3135b, miR-125a-5p, miR-10400-5p, *miR-199b-3p, *miR-199a-3p, miR-9-5p, miR-423-5p, *miR-93-5p, miR-206 | *miR-16-5p, *miR-125b-5p, *miR-199b-3p, *miR-199a-3p, *miR-93-5p, miR-221-3p, miR-432-5p |
| CSPG | *miR-16-5p, miR-3960, miR-3135b, let-7f-5p, miR-10400-5p, *miR-199b-3p, *miR-199a-3p, miR-9-5p, miR-423-5p, *miR-26a-5p | *miR-16-5p, *miR-199b-3p, *miR-199a-3p, *miR-26a-5p, miR-382-5p |
| Iba-1 | miR-3135b, *let-7i-5p, miR-423-5p | *let-7i-5p, miR-23b-3p, miR-23a-3p |
| iNOS | *miR-16-5p, *let-7a-5p, miR-3960, miR-3135b, *miR-29a-3p, let-7f-5p, *let-7b-5p, *let-7i-5p, miR-10400-5p, miR-423-5p, miR-3184-3p, *miR-93-5p, *miR-26a-5p, miR-206 | *miR-16-5p, *let-7a-5p, *miR-29a-3p, *let-7b-5p, * let-7i-5p, *miR-93-5p, *miR-26a-5p, miR-191-5p, miR-23b-3p, miR-432-5p, miR-146a-5p, miR-23a-3p |
| BDNF | *miR-16-5p, *miR-21-5p, *let-7a-5p, miR-3960, miR-3135b, *miR-29a-3p, let-7f-5p, *let-7b-5p, *let-7i-5p, miR-10400-5p, *miR-199b-3p, *miR-199a-3p, miR-9-5p, miR-423-5p, miR-3184-3p, miR-206 | *miR-16-5p, *miR-21-5p, *let-7a-5p, *miR-29a-3p, *let-7b-5p, *let-7i-5p, *miR-199b-3p, *miR-199a-3p, miR-143-3p, miR-221-3p, miR-191-5p, miR-432-5p, miR-382-5p, miR-92a-3p |
| TRPV1 | *miR-16-5p, *let-7a-5p, miR-3960, miR-3135b, *miR-29a-3p, let-7f-5p, *let-7b-5p, *let-7i-5p, miR-10400-5p, miR-9-5p, miR-423-5p | *miR-16-5p, *let-7a-5p, *miR-29a-3p, *let-7b-5p, *let-7i-5p, miR-23b-3p, miR-432-5p, miR-146a-5p, miR-23a-3p |
| Cav3.2 | *miR-16-5p, *miR-125b-5p, *let-7a-5p, miR-3960, miR-3135b, *miR-29a-3p, let-7f-5p, miR-125a-5p, *let-7b-5p, *let-7i-5p, miR-10400-5p, *miR-199b-3p, *miR-199a-3p, miR-423-5p, miR-3184-3p, *miR-93-5p | *miR-16-5p, *miR-125b-5p, *let-7a-5p, miR-92a-3p, * miR-29a-3p, *let-7b-5p, *let-7i-5p, *miR-199b-3p, *miR-199a-3p, *miR-93-5p, miR-143-3p, miR-191-5p, miR-146a-5p, miR-382-5p |
| p75NTR | *miR-16-5p, *let-7a-5p, miR-3960, miR-3135b, miR-125a-5p, *let-7b-5p, miR-10400-5p, miR-423-5p, miR-206 | *miR-16-5p, *let-7a-5p, *let-7b-5p |
| Bax | *let-7a-5p, *miR-29a-3p, let-7f-5p, *let-7b-5p, *let-7i-5p, miR-3184-3p | *let-7a-5p, *miR-29a-3p, *let-7b-5p, *let-7i-5p, miR-432-5p |

HucMSC-EX: human umbilical cord mesenchymal stem cell-derived exosome. *Overlapping miRNAs between source 1 and source 2 HucMSC-EX. Retrieved from two databanks (TargetScan and miRanda).
